# HSCM-Lane: A ResNet-based encoder-decoder architecture with multi-level shifted-window context modeling for pixel-wise lane segmentation

**DOI:** 10.1371/journal.pone.0359959

**Published:** 2026-10-07

**Authors:** Quang Toai Ton, Thanh Hien Vu, Thanh Nguyen Vu, Tuong Le

**Affiliations:** 1 Faculty of Information Technology, HUTECH University, Ho Chi Minh City, Vietnam; 2 Faculty of Information Technology, Ho Chi Minh City University of Foreign Languages - Information Technology, Ho Chi Minh City, Vietnam; 3 Faculty of Information Technology, Ho Chi Minh City University of Industry and Trade, Ho Chi Minh City, Vietnam; Sichuan University, CHINA

## Abstract

Pixel-wise lane segmentation provides dense geometric cues for lane keeping, road-structure inference, and local trajectory planning in advanced driver-assistance systems and autonomous vehicles. The objective is to improve binary lane-mask segmentation under sparse, thin, low-contrast, occluded, and discontinuous lane-marking conditions while retaining a compact real-time configuration. To this end, we propose HSCM-Lane, a ResNet-based encoder-decoder architecture enhanced by a Hierarchical Swin Context Module (HSCM). HSCM organizes shifted-window context modeling at the 1/4, 1/8, and 1/16 encoder feature levels, so that context-enhanced features are propagated through subsequent residual stages and reused by a lightweight decoder with projected static-sum fusion. For backbone-capacity analysis, the architecture is evaluated as HSCM-Lane-S (Small, ResNet18), HSCM-Lane-M (Medium, ResNet34), and HSCM-Lane-L (Large, ResNet50). These configurations differ only in the ResNet backbone, while the HSCM placement, window size, depth, projected static-sum fusion strategy, and Focal-Tversky objective are kept fixed. On the BDD100K validation set, HSCM-Lane-S achieves 34.44% lane-class intersection over union (IoU), 65.00% lane recall, and 82.12% balanced accuracy, compared with 33.00%, 62.48%, and 80.38% for the no-HSCM counterpart. Relative to the no-HSCM counterpart, these values represent gains of 1.44 percentage points in lane IoU, 2.52 points in lane recall, and 1.74 points in balanced accuracy. The lane-IoU gain corresponds to a 4.36% relative improvement. Scaling the backbone yields 34.86% lane IoU for HSCM-Lane-M and 34.98% lane IoU for HSCM-Lane-L, showing a gradual quality-cost trade-off. Under a derived pixel-level out-of-domain evaluation protocol on TuSimple, HSCM-Lane-S, HSCM-Lane-M, and HSCM-Lane-L achieve 27.2%, 27.6%, and 27.6% lane IoU, respectively, without target-domain fine-tuning. These results indicate that multi-level shifted-window context modeling improves the no-HSCM counterpart and yields competitive lane-mask segmentation results under these protocols. Future work will address temporal, instance-level, multimodal, planning, and safety validation.

## Introduction

Pixel-wise lane segmentation predicts a binary mask in which each pixel is assigned to either the background class or the lane-marking class. The resulting dense geometry can support lane keeping, road-structure inference, and local trajectory planning. This formulation differs from instance-level lane detection and row-wise lane-coordinate prediction, which produce separate lane instances, fitted lane curves, or sparse lane coordinates rather than a single binary mask.

The task is difficult because lane markings are thin, elongated, low-contrast, highly sparse, and perspective-dependent. Far-field markings may occupy only a few pixels, while nearby markings can be occluded by vehicles, interrupted by worn paint, or degraded by shadows, road-surface reflections, nighttime illumination, and adverse weather. SCNN [1] highlighted the importance of spatial relationships for lanes with weak appearance cues, while ENet-SAD [2] emphasized that the supervisory signal from lane annotations is sparse and subtle. A suitable model must therefore preserve local geometry while using broader context to maintain continuity across weak or discontinuous lane regions.

Lane-specific methods address these challenges through different output representations. LaneNet [3] predicts lane instances, SCNN [1] propagates information across feature maps, ENet-SAD [2] transfers self-attention knowledge to a lightweight network, and LaneAF [4] combines a binary mask with affinity fields for lane grouping. These methods improve structural reasoning, but their instance outputs, auxiliary fields, or message-passing designs are not identical to the compact binary lane-mask setting studied here.

Multitask driving-perception systems provide broader scene context by sharing features across tasks. YOLOP [5] jointly performs object detection, drivable-area segmentation, and lane-line segmentation, while TwinLiteNet [6] and TwinLiteNet+ [7] emphasize lightweight dual-task segmentation. Such systems offer system-level efficiency, but shared representations may involve task-level trade-offs; consequently, the lane branch should also be evaluated as a dedicated segmentation objective.

General dense-prediction architectures provide complementary design principles. DeepLabV3+ [8] combines contextual encoding with boundary refinement, SegFormer [9] couples hierarchical features with a lightweight decoder, Swin Transformer [10] exchanges information across shifted local windows, and ResNet [11] provides stable residual optimization with a strong convolutional local bias. The design question addressed in this work is therefore how to introduce multi-level context without discarding the fine spatial information required by thin lane markings.

To address this problem, we propose HSCM-Lane, a ResNet-based encoder-decoder for pixel-wise binary lane segmentation. Its Hierarchical Swin Context Module (HSCM) applies shifted-window context modeling at the 1/4, 1/8, and 1/16 encoder levels. The refined features are propagated through later residual stages and reused by a lightweight decoder with projected static-sum fusion. HSCM is thus a task-specific organization of existing shifted-window operations, not a new generic self-attention operator or Transformer backbone. The selected levels span complementary roles: 1/4 retains fine lane geometry, 1/8 balances detail and context, and 1/16 provides broader road-layout information. The 1/2 stem remains a direct decoder skip to avoid high attention cost at that resolution, while the 1/32 stage is omitted to reduce the loss of thin far-field evidence caused by additional downsampling.

The same architecture is evaluated at three backbone capacities: HSCM-Lane-S (ResNet18), HSCM-Lane-M (ResNet34), and HSCM-Lane-L (ResNet50). BDD100K is the main training and validation benchmark; TuSimple is used only for out-of-domain testing under the derived pixel-level protocol. Component ablations use HSCM-Lane-S, while the M and L variants are used to examine the backbone-capacity trade-off.

The main contributions are as follows.

- First, HSCM-Lane integrates shifted-window context modeling into the 1/4, 1/8, and 1/16 levels of a ResNet encoder, propagating the refined features through the encoder hierarchy and reusing them in the decoder.
- Second, the model uses a compact decoder with projected static-sum fusion and a combined Focal-Tversky objective to reconstruct sparse binary lane masks.
- Third, controlled BDD100K experiments evaluate the HSCM contribution, placement, window size, depth, decoder fusion, loss composition, backbone capacity, and five-seed stability.
- Fourth, the study reports BDD100K comparisons, a derived pixel-level out-of-domain evaluation on TuSimple, condition-wise analyses, and qualitative comparisons of the HSCM contribution and cross-domain behavior.

The remainder of the paper reviews related work, presents HSCM-Lane, reports the experimental evaluation, discusses trade-offs and limitations, and concludes the study.

## Related work

Related work is organized into lane-specific methods, general dense-prediction architectures, and lightweight or multitask driving-perception systems. The final subsection then positions HSCM-Lane and distinguishes direct quantitative baselines from architectural design references because these methods do not all produce the same binary lane-mask output.

### Lane-specific methods at pixel level and instance level

Within the lane-specific line of research, previous studies have approached lane perception at two closely related but not entirely identical levels: the pixel level and the instance level. LaneNet [3] formulates the problem within an instance segmentation framework by treating each lane as a separate instance. SCNN [1] shows that, for long, thin, and continuous structures such as lane markings, propagating information directly within the feature-map space plays a more important role than merely stacking deeper convolutional layers. ENet-SAD [2] follows a lightweight semantic segmentation approach and uses self-attention distillation to improve contextual representations at low computational cost. LaneAF [4] jointly predicts a binary lane mask and per-pixel affinity fields, thereby supporting the grouping of pixels into individual lanes in a more explicitly structured manner. IALaneNet [12] further exploits the interaction between lane area and lane marking, showing that modeling relationships among outputs with strong geometric correlation can also improve segmentation quality.

More recently, AFLaneNet [13] and the two-branch instance segmentation method of Wang et al. [14] have further advanced the lane-specific direction at the instance level. Although presented under the name of lane detection, both studies are based on an instance segmentation framework and emphasize the role of attention, multi-level fusion, and multi-scale representations in maintaining lane continuity under complex observation conditions. In this paper, we regard such studies as directly relevant design references because they address lane perception through pixel-level or instance-level representations. However, due to differences in the final output format and evaluation protocol, these methods are not used as direct one-to-one quantitative baselines for the pixel-wise binary lane segmentation problem considered in the present study. They are instead used to motivate the need for spatial-context modeling and geometric-continuity preservation in lane perception.

Overall, this body of work indicates that lane segmentation cannot be handled effectively through independent per-pixel classification alone because it requires specialized mechanisms to maintain geometric continuity, long-range spatial relationships, and consistency across different parts of a lane.

### General semantic segmentation architectures and hierarchical representations

In parallel with lane-specific models, general dense prediction architectures provide several important design cues for pixel-wise lane segmentation. DeepLabV3+ [8] demonstrates the effectiveness of combining multi-scale context with a compact decoder to refine segmentation boundaries. SegFormer [9] shows that a hierarchical Transformer encoder can be combined effectively with a lightweight decoder to produce strong multi-level representations for semantic segmentation. Swin Transformer [10] introduces shifted-window attention, in which self-attention is computed within local windows and window partitions are shifted between consecutive blocks to enable cross-window information exchange at moderate computational cost. This mechanism is relevant to lane segmentation because lane markings may cross local window boundaries and require contextual support from neighboring road regions. At the same time, ResNet [11] remains a reliable foundation because residual learning stabilizes optimization and convolutional layers preserve local inductive bias, which is useful for thin lane-marking details. Unlike the lane-specific methods discussed above, the models in this group are not designed specifically for lanes, but they provide general principles for hierarchical representation, context modeling, and segmentation-boundary recovery. From a design perspective, this body of work suggests an appropriate direction for lane segmentation: retaining a stable convolutional backbone to preserve the local geometric detail of thin lane markings, while introducing multi-level shifted-window context modeling at intermediate encoder feature levels to support spatial continuity across weak, occluded, or discontinuous lane regions.

In this paper, shifted-window attention is used as a context-modeling principle rather than as a claim of a new generic Transformer operator. The proposed HSCM is inserted into a ResNet encoder hierarchy at the 1/4, 1/8, and 1/16 feature levels, so that context-enhanced features are propagated through subsequent residual stages and reused by the decoder. This design differs from simply appending an attention block at the deepest encoder level or relying only on decoder-side refinement.

### Lightweight and multitask driving perception systems

Within the context of unified perception for autonomous driving, BDD100K [15] has established a heterogeneous multitask benchmark in which the lane branch is learned jointly with drivable-area segmentation, object detection, and other contextual signals. YOLOP [5] is a representative milestone in this direction, employing a shared encoder and task-specific decoders. Subsequently, HybridNets [16], CenterPNets [17], Mobip [18] based on MobileNetV2 [19], and Sparse U-PDP [20] have further developed panoptic driving perception architectures with different priorities regarding the degree of feature sharing, context utilization, and inference cost.

More recently, lightweight models such as TwinLiteNet [6], TwinLiteNet+ [7], and TriLiteNet [21], together with context-enhanced multitask variants such as YOLOPv3 [22], UF-Net [23], and RLSNet [24], have continued to emphasize the need to balance lane-branch quality, architectural coherence, and practical deployability. TwinMixing [25] further follows this lightweight multitask direction by targeting drivable-area and lane-line segmentation with feature interaction and efficient mixing mechanisms. Because its lane branch is directly related to lane segmentation, TwinMixing is relevant as a multitask comparison reference, although differences in task setting, implementation, and evaluation protocol should still be considered when interpreting quantitative comparisons. Unified perception models that do not directly target binary lane-mask segmentation are also useful for system-level context. UniPercepNet-S [26], for example, is a lightweight dual-task framework for real-time object detection and instance segmentation. Although it reflects the broader trend toward compact unified perception, its output format is different from the binary lane-mask output considered in this paper. Therefore, UniPercepNet-S is discussed as related system-level context rather than used as a direct quantitative baseline for lane IoU comparison.

Among these systems, RLSNet jointly learns drivable-area segmentation, lane-line detection, and scene identification, showing that cross-task information can constrain lane perception in complex scenes. However, system-level multitask benefits do not ensure that the shared representation is optimal for a binary lane-mask objective. We therefore use multitask studies as system-level context and feature-sharing references, while HSCM-Lane remains a specialized single-task segmentation model.

Beyond shared local perception tasks, recent work has begun to connect onboard observations with global navigation context. NavigScene [27] pairs local multi-view sensor inputs with natural-language navigation guidance and studies navigation-guided reasoning, preference optimization, and vision-language-action feature fusion for perception, prediction, and planning. This direction is complementary to HSCM-Lane. The proposed model produces a local pixel-wise lane mask and does not model beyond-visual-range navigation intent, but its geometry-focused output can serve as one local cue within a larger navigation-guided perception and planning pipeline.

### Positioning of HSCM-Lane

The reviewed literature suggests that effective pixel-wise lane segmentation requires four complementary properties. First, the model must preserve local geometric detail because lane markings are thin, sparse, and boundary-sensitive. Second, it must model broader spatial context because lane markings are elongated, partially discontinuous, and frequently degraded by occlusion, weak contrast, or illumination variation. Third, it must recover spatial resolution through an efficient decoder because the final output is a dense binary mask. Fourth, its performance should be evaluated with metrics and protocols that focus on the lane class rather than the dominant background class.

HSCM-Lane follows these requirements by combining a ResNet-based encoder, multi-level shifted-window context modeling, projected static-sum decoder fusion, and a Focal-Tversky objective. The novelty of HSCM-Lane is not the invention of a new attention operator or a new generic Transformer backbone. Instead, the contribution lies in the task-specific organization of HSCM at multiple intermediate levels of a ResNet encoder for pixel-wise lane segmentation. In this design, context-enhanced features are generated before decoding, propagated through later residual stages, and reused by the decoder through skip connections.

This positioning also motivates the subsequent experimental design. Rather than relying only on comparisons with external baselines, the paper evaluates HSCM-Lane through controlled ablations, including the presence of HSCM, HSCM placement, window size, HSCM depth, decoder fusion strategy, loss components, ResNet backbone capacity, and repeated-run statistics. This design is intended to test whether the proposed organization of shifted-window context modeling provides a measurable benefit under controlled conditions.

## Proposed method

Throughout this section, the subscripts 1/2, 1/4, 1/8, and 1/16 denote the feature-resolution ratios relative to the input image. Feature tensors are denoted by bold letters, whereas learnable transformations are denoted by calligraphic symbols or upright operators. This notation clarifies the role of each feature level in the hierarchical architecture of the model. Here, HSCM denotes the Hierarchical Swin Context Module. In this paper, “hierarchical” refers to the placement of shifted-window context modeling at multiple encoder resolutions, namely the 1/4, 1/8, and 1/16 feature levels, and to the propagation of context-enhanced features through subsequent residual stages. It does not refer to a new generic Transformer backbone or a new self-attention operator.

### Problem formulation and overall architecture

In this study, the lane segmentation problem is formulated as a pixel-wise binary semantic segmentation task. Given an input image $𝐈\in R^{\text{3}\times H\times W}$, where H is the image height and W is the image width, the objective of the model is to predict a logits tensor $𝐙\in R^{\text{2}\times H\times W}$, in which the two output channels correspond to the background class and the lane class. The overall mapping from the input image to the segmentation logits is written as


$$\begin{array}{c} 𝐙=f_{\theta }\left(𝐈\right),\;𝐈\in R^{\text{3}\times H\times W},\;𝐙\in R^{\text{2}\times H\times W} \end{array}$$ (1)

where $f_{\theta }(\cdot )$ is the HSCM-Lane model with parameter set θ. According to Eq. (1), the output of the model is a pixel-wise classification map, so the model must simultaneously learn the semantics of the lane region and preserve the thin, elongated, and continuous geometry of lane markings.

To meet this requirement, HSCM-Lane is constructed as an encoder-decoder architecture, as shown in Fig 1. The encoder is responsible for hierarchical feature extraction and spatial context enhancement, whereas the decoder progressively restores spatial resolution to reconstruct the lane mask. Specifically, HSCM-Lane uses a ResNet backbone to extract hierarchical convolutional features. The architectural description in this section focuses on the common tensor flow shared by the encoder, HSCM, decoder, and segmentation head. The ResNet backbone may be instantiated with different capacities in the experiments, but the exported feature interface passed to HSCM, and the decoder is kept fixed. In the experimental evaluation, these backbone configurations are denoted as HSCM-Lane-S (Small, ResNet18), HSCM-Lane-M (Medium, ResNet34), and HSCM-Lane-L (Large, ResNet50). Based on the context-enhanced features, a multi-level decoder with a projected static-sum fusion mechanism is used to fuse deep and shallow features.

**Fig 1:**
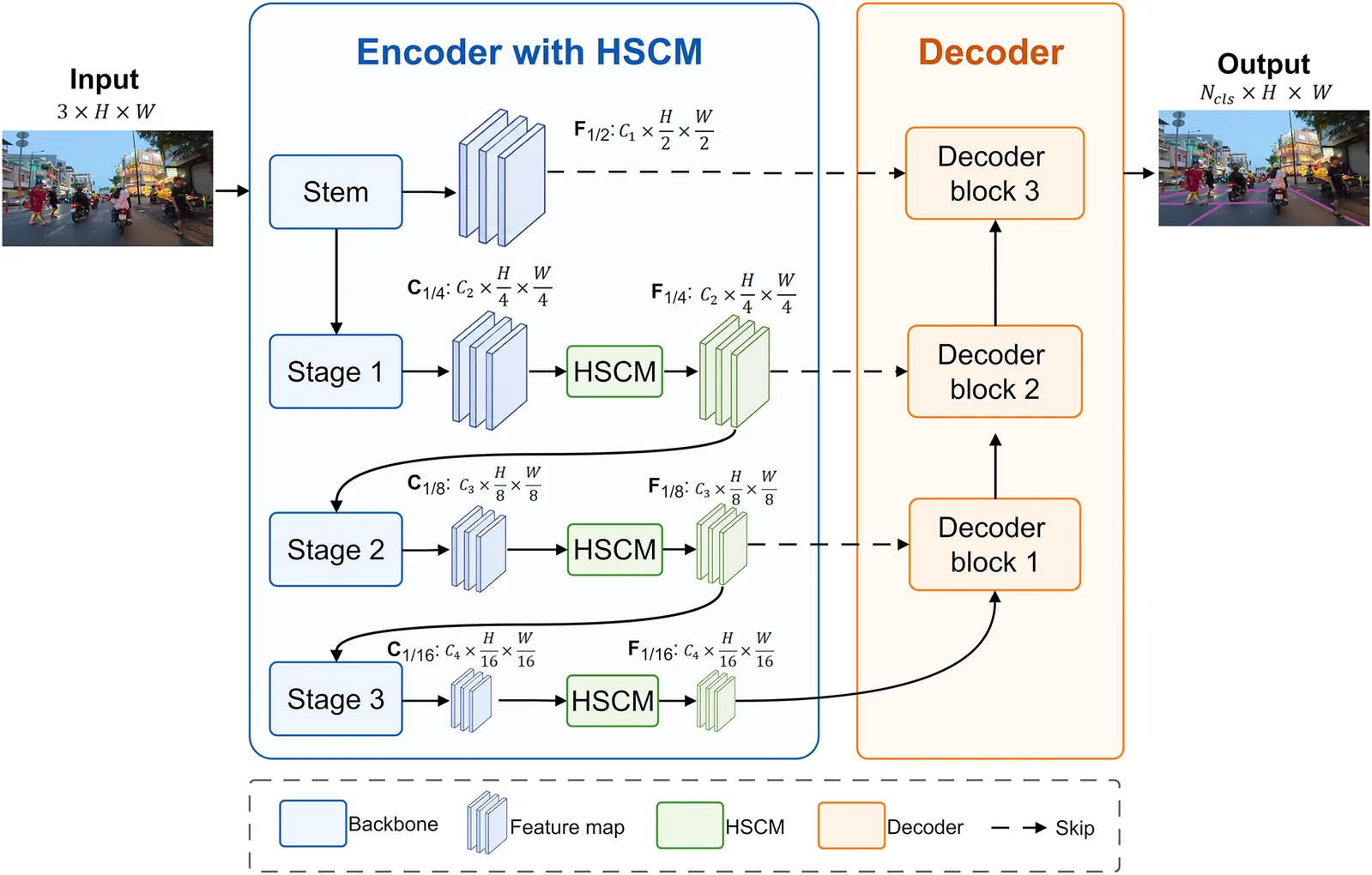
Overall architecture of HSCM-Lane with a ResNet backbone. The encoder extracts multi-resolution ResNet features, HSCM enhances the 1/4, 1/8, and 1/16 feature levels using shifted-window context modeling, and the lightweight decoder progressively recovers the binary lane mask through multi-level skip fusion. The S/M/L configurations evaluated in this study follow the same block diagram and differ only in ResNet backbone capacity. The road-scene photograph was taken by the first author and is provided for publication under the CC BY 4.0 license. The model-output overlay and all schematic elements were generated by the authors.

Unless otherwise stated, Fig 1 and Table 1 describe the decoder-facing tensor flow of HSCM-Lane. The spatial sizes are determined by the common ResNet hierarchy for the 384 × 640 input. The channel numbers denote the exported feature channels passed to HSCM and the decoder. When a higher-capacity ResNet backbone produces different raw stage channels, projection layers align them to the same exported channel interface before HSCM and decoder fusion.

**Table 1:** Decoder-facing tensor flow of HSCM-Lane for an input size of 384 × 640.

Component	Output	Spatial size	Exported Channels	Main operation	Decoder use
Input	𝐈	384 × 640	3	RGB image	–
Stem	${𝐅}_{\text{1}/\text{2}}$	192 × 320	64	7 × 7 Conv, BN, ReLU	Skip to decoder block 3
ResNet Stage 1 + HSCM	${𝐅}_{\text{1}/\text{4}}$	96 × 160	64	residual stage + HSCM	Skip to decoder block 2
ResNet Stage 2 + HSCM	${𝐅}_{\text{1}/\text{8}}$	48 × 80	128	residual stage + HSCM	Skip to decoder block 1
ResNet Stage 3 + HSCM	${𝐅}_{\text{1}/\text{16}}$	24 × 40	256	residual stage + HSCM	Initial decoder input
Decoder block 1	${𝐃}_{\text{1}/\text{8}}$	48 × 80	128	upsample + projected static-sum fusion	Next decoder block
Decoder block 2	${𝐃}_{\text{1}/\text{4}}$	96 × 160	64	upsample + projected static-sum fusion	Next decoder block
Decoder block 3	${𝐃}_{\text{1}/\text{2}}$	192 × 320	64	upsample + projected static-sum fusion	Segmentation head
Segmentation head	𝐙	384 × 640	2	3 × 3 Conv-BN-SiLU, 1 × 1 Conv, bilinear upsampling	Final logits

Table 1 summarizes the decoder-facing tensor flow of HSCM-Lane at the input size used in this study. The four main encoder feature levels are denoted by ${𝐅}_{\text{1}/\text{2}}$, ${𝐅}_{\text{1}/\text{4}}$, ${𝐅}_{\text{1}/\text{8}}$, and ${𝐅}_{\text{1}/\text{1}\text{6}}$, with spatial sizes 192 × 320, 96 × 160, 48 × 80, and 24 × 40, respectively. These features are exposed to HSCM and the decoder with channel dimensions 64, 64, 128, and 256. For different ResNet backbone capacities, projection layers are used when needed to preserve this common exported feature interface.

### ResNet backbone with multi-level HSCM placement

The encoder is built on a ResNet backbone [11]. It uses the stem and the first three residual stages up to the 1/16 scale, while the 1/32 stage is not used because lane markings are thin and far-field lane pixels may be weakened by excessive downsampling. The exported feature channels passed to HSCM, and the decoder are fixed to 64/128/256 at the 1/4, 1/8, and 1/16 levels, respectively. In the experiments, ResNet18, ResNet34, and ResNet50 are used as the S, M, and L backbone configurations, while the same spatial hierarchy, HSCM placement, and decoder-facing channel interface are preserved. The ResNet encoder is selected for two reasons. First, the residual learning mechanism provides stable optimization, which is appropriate when the encoder is further enhanced with shifted-window context modeling. Second, the convolutional layers of the ResNet encoder preserve a strong local inductive bias, which is highly suitable for representing thin, narrow, and elongated lane markings. However, lane segmentation requires not only local recognition but also the ability to connect lane segments with geometric relationships over longer distances, especially in regions affected by occlusion, reflections, or contrast degradation. Therefore, the intermediate features of the ResNet encoder are further refined by HSCM before being passed to deeper layers.

Let S(·) denote the ResNet stem, P(·) denote max-pooling, and let $R_{\text{1}/\text{4}},\;R_{\text{1}/\text{8}}$, and $R_{\text{1}/\text{1}\text{6}}$ denote the first three residual stages of the ResNet encoder. The encoder flow is defined as


$$\begin{array}{c} \left\{ \begin{array}{c} 𝐅\text{1}/\text{2}\;=S\left(𝐈\right)\;\\ {𝐂}_{\text{1}/\text{4}}\;=R_{\text{1}/\text{4}}\left(P({𝐅}_{\text{1}/\text{2}})\right),\;\;\;\;\;\;{𝐅}_{\text{1}/\text{4}}=H_{\text{1}/\text{4}}\left({𝐂}_{\text{1}/\text{4}}\right)\;\\ {𝐂}_{\text{1}/\text{8}}\;=R_{\text{1}/\text{8}}\left({𝐅}_{\text{1}/\text{4}}\right),\;\;\;\;\;\;\;\;\;\;{𝐅}_{\text{1}/\text{8}}\;=H_{\text{1}/\text{8}}\left({𝐂}_{\text{1}/\text{8}}\right)\;\\ {𝐂}_{\text{1}/\text{1}\text{6}}\;=R_{\text{1}/\text{1}\text{6}}\left({𝐅}_{\text{1}/\text{8}}\right),\;\;\;{𝐅}_{\text{1}/\text{1}\text{6}}=H_{\text{1}/\text{1}\text{6}}\left({𝐂}_{\text{1}/\text{1}\text{6}}\right)\; \end{array}\right. \end{array}$$ (2)

In Eq. (2), ${𝐂}_{\text{1}/\text{4}}$, ${𝐂}_{\text{1}/\text{8}}$ and ${𝐂}_{\text{1}/\text{1}\text{6}}$ are the direct outputs of the residual stages, whereas ${𝐅}_{\text{1}/\text{4}}$, ${𝐅}_{\text{1}/\text{8}}$ and ${𝐅}_{\text{1}/\text{1}\text{6}}$ are the features after HSCM-based context refinement. An important property of this flow is that ${𝐅}_{\text{1}/\text{4}}$ and ${𝐅}_{\text{1}/\text{8}}$ are not only passed to the decoder as skip features but are also fed into subsequent residual stages. Therefore, HSCM is not an independent post-processing block because it directly participates in the formation of hierarchical encoder representations. The structure of one HSCM stage is summarized in Fig 2.

**Fig 2:**
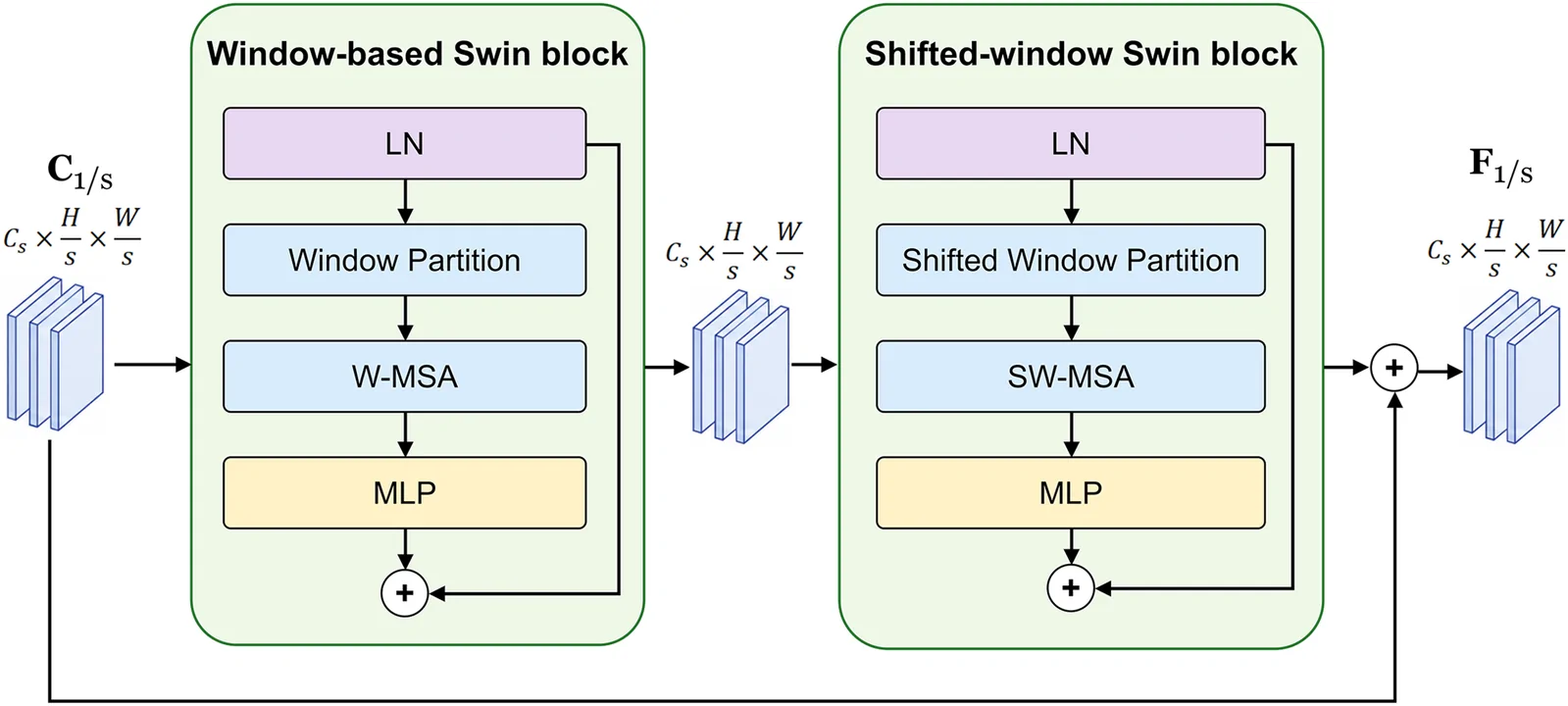
Structure of one HSCM stage. Each HSCM stage contains a window-based Swin block followed by a shifted-window Swin block. The first block performs local window attention, whereas the second block enables cross-window information exchange through shifted-window partitioning.

Within HSCM, we use Swin-style shifted-window context modeling to enhance multi-scale CNN features. The module combines self-attention within local windows with a shifted-window mechanism between consecutive blocks, thereby allowing neighboring feature regions to exchange information while avoiding global self-attention over the entire feature map. In Fig 2, layer normalization (LN) normalizes each token representation, the multilayer perceptron (MLP) provides channel-wise transformation, window-based multi-head self-attention (W-MSA) operates within non-overlapping windows and shifted-window multi-head self-attention (SW-MSA) uses the shifted partition to exchange information across neighboring windows.

Structurally, HSCM consists of three stages, $H_{\text{1}/\text{4}}$, $H_{\text{1}/\text{8}}$, and $H_{\text{1}/\text{1}\text{6}}$, operating at the three intermediate feature levels of the backbone. Each stage has depth 2 and uses a window size of M=8. To avoid ambiguity, we use s to denote the downsampling factor rather than the feature-resolution ratio. Thus, s∈{4, 8, 16}, and the corresponding feature level is 1/s with spatial size H/s×W/s. As illustrated in Fig 2, one HSCM stage contains a window-based Swin block followed by a shifted-window Swin block. For s∈{4, 8, 16}, the HSCM stage at the 1/s level is written as


$$\begin{array}{c} H_{\text{1}/s}\left({𝐂}_{\text{1}/s}\right)=T_{\text{1}/s}^{SW}\left(T_{\text{1}/s}^{W}\left({𝐂}_{\text{1}/s}\right)\right) \end{array}$$ (3)

where $T_{\text{1}/s}^{\text{W}}(\cdot )$ denotes a Swin-style block without window shifting at the 1/s feature level, and $T_{\text{1}/s}^{\text{SW}}(\cdot )$ denotes a Swin-style block with shifted windows. The first block partitions the feature map into non-overlapping local windows and applies window-based multi-head self-attention within each window. The second block shifts the window partition before attention is computed, enabling cross-window information exchange. After channel alignment to the exported feature interface, the numbers of attention heads are set to 4, 8, and 8 at the 1/4, 1/8, and 1/16 levels, respectively.

The selected HSCM levels cover complementary representation scales while controlling computational cost. At the 1/4 scale, HSCM preserves the spatial detail needed for thin lane boundaries. At the 1/8 scale, it balances geometric detail with broader road context. At the 1/16 scale, it provides higher-level road-layout information. The 1/2 stem remains a direct decoder skip feature rather than being processed by HSCM because its larger spatial size would substantially increase the cost of window attention. The 1/32 stage is omitted because additional downsampling can weaken thin far-field lane evidence. At each selected level, a window-based block first models local relations, and the following shifted-window block enables information exchange across the preceding window boundaries. The single-level and multi-level placement choices are evaluated under fixed settings in the controlled ablation study. Therefore, the contribution of HSCM lies in the task-specific placement and propagation of shifted-window context modeling inside the ResNet encoder hierarchy, not in the invention of a new self-attention operator.

### Multi-level decoder with projected static-sum fusion

After the encoder, the model obtains four feature levels, ${𝐅}_{\text{1}/\text{2}}$, ${𝐅}_{\text{1}/\text{4}}$, ${𝐅}_{\text{1}/\text{8}}$, and ${𝐅}_{\text{1}/\text{1}\text{6}}$, as described above. The decoder is responsible for transforming these representations into a pixel-wise lane mask. Unlike the encoder, the decoder is designed to be compact and stable, because most of the context-modeling capacity has already been introduced in HSCM. Accordingly, the main role of the decoder is to progressively restore spatial resolution and combine deep features with shallow features. The projected static-sum fusion design inside each decoder block is summarized in Fig 3.

**Fig 3:**
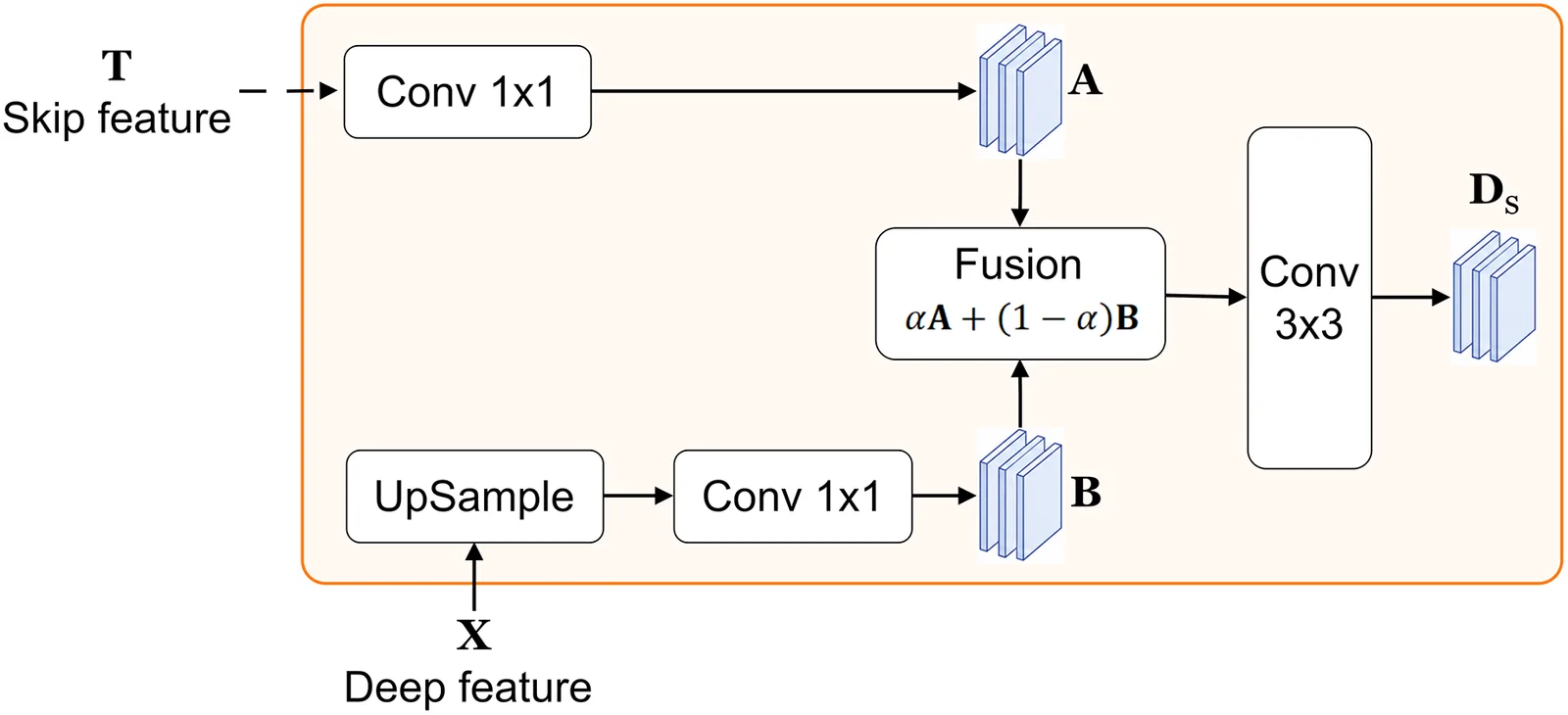
Decoder block with projected static-sum fusion. The skip feature is projected by a 1 × 1 convolution, the deep feature is upsampled and projected by another 1 × 1 convolution, and the two aligned branches are fused before local 3 × 3 refinement.

Operationally, the decoder contains three decoder blocks. Decoder block 1 upsamples the 1/16 feature ${𝐅}_{\text{1}/\text{1}\text{6}}$ to the 1/8 resolution and fuses it with ${𝐅}_{\text{1}/\text{8}}$, producing ${𝐃}_{\text{1}/\text{8}}$. Decoder block 2 upsamples ${𝐃}_{\text{1}/\text{8}}\;$to the 1/4 resolution and fuses it with ${𝐅}_{\text{1}/\text{4}}$, producing ${𝐃}_{\text{1}/\text{4}}$. Decoder block 3 upsamples ${𝐃}_{\text{1}/\text{4}}$ to the 1/2 resolution and fuses it with the shallow stem feature ${𝐅}_{\text{1}/\text{2}}$, producing ${𝐃}_{\text{1}/\text{2}}$. This final skip connection gives the decoder direct access to fine spatial details before the segmentation head.

Each decoder block uses the projected static-sum fusion shown in Fig 3. The deep branch is first bilinearly upsampled and projected by a 1 × 1 convolution. The skip branch is projected by another 1 × 1 convolution so that both branches have the same channel dimension. The two aligned branches are then combined using a fixed mixing coefficient α=0.5, and the fused feature is refined by a local 3 × 3 Conv-BN-SiLU block. This design keeps the decoder lightweight while allowing deep semantic information and shallow spatial detail to interact at each resolution level.

From a representational perspective, the upsampled deep branch carries more semantic information because it originates from a deeper layer, whereas the skip branch better preserves positional and boundary information because it has higher spatial resolution. By repeatedly applying this fusion strategy across the three decoder blocks, the model progressively transitions from a semantics-rich representation to a geometry-rich representation. Specifically, ${𝐃}_{\text{1}/\text{8}}$ mainly inherits semantics from ${𝐅}_{\text{1}/\text{1}\text{6}}$ but already begins to recover spatial structure through ${𝐅}_{\text{1}/\text{8}}$; ${𝐃}_{\text{1}/\text{4}}$ is further supplemented with boundary and positional information from ${𝐅}_{\text{1}/\text{4}}$; and ${𝐃}_{\text{1}/\text{2}}$ directly accesses the best local information from ${𝐅}_{\text{1}/\text{2}}$, thereby forming the basis for the subsequent mapping into class space.

The projected static-sum fusion design is evaluated against concatenation-based and gated fusion variants in the ablation study.

### Segmentation head and final output

After the three decoding steps, the model obtains the final decoder feature ${𝐃}_{\text{1}/\text{2}}\in R^{\text{6}\text{4}\times H/\text{2}\times W/\text{2}}$. On this tensor, a lightweight segmentation head is used to transform the representation from feature space to class space. The head structure is kept minimal to avoid increasing inference cost, because most of the model’s representational capacity already lies in the encoder and the multi-level decoder.

The segmentation head applies a 3 × 3 Conv-BN-SiLU block to ${𝐃}_{\text{1}/\text{2}}$, reducing the feature dimension from 64 to 32 channels. A final 1 × 1 convolution then maps the feature to two logits channels, corresponding to background and lane. The logits are bilinearly upsampled to the input resolution, and the final label at each pixel is selected as the class with the larger logit value. Overall, Figs 1 and 3 show that the decoder and segmentation head form a compact reconstruction pipeline: spatial resolution is progressively recovered through multi-level skip fusion, while the final prediction head remains lightweight.

### Loss function

To address severe class imbalance, we combine Focal loss [28] with a focal Tversky loss variant [29,30]. The segmentation head produces the final logits tensor 𝐙 for the two classes, background and lane. During training and evaluation, to match the ground truth of the lane branch with size 360×640, we crop 12 pixels from the top edge and 12 pixels from the bottom edge along the vertical dimension of the logits tensor 𝐙 before computing the loss and the evaluation metrics. Let $\tilde{𝐙}$ denote the logits tensor after cropping. All loss terms below are computed on $\tilde{𝐙}$. Let N be the total number of pixels in the mini-batch after cropping, let $z_{i,c}$ be the logit of pixel i for class c∈{0,1}, and let $y_{i,c}\in \{\text{0},\text{1}\}$ be the corresponding one-hot label.

To handle the severe class imbalance between background and lane, we use Focal loss on each logit channel in a one-vs-rest scheme. Let $q_{i,c}=sigmoid\left(z_{i,c}\right),\;{\text{𝒾}}_{i,c}^{BCE}=-y_{i,c}\text{log}q_{i,c}-\left(\text{1}-y_{i,c}\right)\text{log}\left(\text{1}-q_{i,c}\right),\;p_{t,i,c}=e^{-{\text{𝒾}}_{i,c}^{BCE}}=y_{i,c}q_{i,c}+\;\left(\text{1}-y_{i,c}\right)\left(\text{1}-q_{i,c}\right)$, $\begin{array}{c} \alpha_{i,c}=\alpha_{\text{3}}y_{i,c}+\left(\text{1}-\alpha_{\text{3}}\right)\left(\text{1}-y_{i,c}\right). \end{array}$ Then


$$\begin{array}{c} L_{focal}=\frac{\text{1}}{N}\sum_{c=\text{0}}^{\text{1}}\sum_{i=\text{1}}^{N}\alpha_{i,c}{\left(\text{1}-\;p_{t,i,c}\right)}^{\gamma_{\text{3}}}{\text{𝒾}}_{i,c}^{BCE} \end{array}$$ (4)

According to Eq. (4), pixels that have already been classified correctly with high confidence receive smaller weights, whereas difficult pixels, especially in faint, thin, or occluded lane regions, contribute more strongly to the optimization gradient. In the experimental setting of this paper, the corresponding coefficients are set to $\alpha_{\text{3}}=\text{0}.\text{2}\text{5}$ and $\gamma_{\text{3}}=\text{2}.\text{0}$.

In addition, to directly optimize the overlap quality between the predicted mask and the reference mask, we use a focal Tversky loss variant, in which the aggregated Tversky error term is raised to the power $\gamma_{\text{2}}$. Unlike the focal term, the class probabilities here are computed by softmax along the class dimension. Let $s_{i,c}=\text{softmax}({𝐳}_{i}{)}_{c}$, where ${𝐳}_{i}=(z_{i,\text{0}},z_{i,\text{1}})$ is the logits vector at pixel i. For each class c, we have ${TP}_{c}=\sum_{i=\text{1}}^{N}s_{i,c}y_{i,c}$, ${FP}_{c}=\sum_{i=\text{1}}^{N}s_{i,c}\left(\text{1}-y_{i,c}\right)$, and ${FN}_{c}=\sum_{i=\text{1}}^{N}\left(\text{1}-s_{i,c}\right)y_{i,c}$. The Tversky index of class c is then defined as $T_{c}=\frac{{TP}_{c}}{\text{max}\left({TP}_{c}+\alpha_{\text{2}}\;{FP}_{c}+\beta_{\text{2}}{FN}_{c},\;\varepsilon \right)}$.

To prevent a class that does not appear in the mini-batch ground truth from introducing an undesired contribution to the loss function, we use an indicator variable $m_{c}\in \{\text{0},\text{1}\}$, where $m_{c}=\text{1}$ if class c is present and $m_{c}=\text{0}$ otherwise. In this way, an absent class is set to zero at the class-term level. After this step, the class terms are still aggregated by averaging over the fixed number of classes C. Therefore, the absent class does not contribute to the term $\left(\text{1}-T_{c}\right)$, but the final loss value remains normalized by C. Accordingly, the modified Tversky loss is defined as


$$\begin{array}{c} L_{\text{Tversky}}={\left[\frac{\text{1}}{C}\sum_{c=\text{0}}^{C-\text{1}}m_{c}\left(\text{1}-T_{c}\right)\right]}^{\gamma_{\text{2}}},\;C=\text{2} \end{array}$$ (5)

In the present task, C=2 corresponds to the two classes, background and lane. This formulation removes the direct contribution of an absent class while maintaining consistent normalization across the class space. This design intentionally preserves a fixed loss scale across batches, even when one class is absent. In Eq. (5), $\alpha_{\text{2}}$ is the false-positive penalty coefficient, $\beta_{\text{2}}$ is the false-negative penalty coefficient, with $\beta_{\text{2}}=\text{1}-\alpha_{\text{2}}$, and ε is a small constant for numerical stability. In the configuration used in this paper, $\alpha_{\text{2}}=\text{0}.\text{9}$, $\beta_{\text{2}}=\text{0}.\text{1}$, and $\gamma_{\text{2}}=\text{1}.\text{3}\text{3}\text{3}\text{3}$. This choice helps the model effectively control the overprediction of lane pixels on the road background while still maintaining pixel-level overlap quality.

From Eqs. (4) and (5), the final objective function of the model is written as


$$\begin{array}{c} L=\lambda_{focal}L_{focal}+\lambda_{\text{Tversky}}L_{\text{Tversky}} \end{array}$$ (6)

In the reported experiments, $\lambda_{focal}=\lambda_{\text{Tversky}}=\text{1}.\text{0}$. Thus, the optimization process simultaneously leverages the advantage of Focal loss in handling class imbalance and that of Tversky loss in optimizing segmentation overlap quality.

## Experiments and evaluation

This section reports the datasets, metrics, implementation, controlled ablations, comparative results, out-of-domain evaluation, condition-wise analysis, and qualitative results. HSCM-Lane-S, HSCM-Lane-M, and HSCM-Lane-L use ResNet18, ResNet34, and ResNet50, respectively; the HSCM, decoder, loss, and training settings are otherwise fixed. Within the controlled ablation study, HSCM-Lane-S is used for component ablations, whereas HSCM-Lane-M and HSCM-Lane-L are used for the backbone-capacity analysis. All three variants are subsequently included in the comparative and cross-dataset evaluations. The no-HSCM ResNet18 baseline and the three HSCM-Lane variants are evaluated over five seeds and reported as mean ± standard deviation. The remaining component ablations are representative controlled runs under fixed settings.

### Datasets and evaluation protocol

Table 2 summarizes the data splits actually used in this study. Throughout the paper, BDD100K [15] is used as the main benchmark for both training and evaluation, whereas TuSimple [31] is used only as an out-of-domain test set to analyze the generalization capability of the model.

**Table 2:** Summary of the data splits used in this study.

Dataset	Training split used	Evaluation split used
TuSimple	Not used	2,782 (test)
BDD100K	70,000	10,000 (val)

We chose BDD100K as the central dataset because it was collected from vehicle-mounted cameras under diverse weather conditions, times of day, and street-scene contexts, making it suitable for evaluating lane-mask segmentation under varied validation conditions. The study follows the standard BDD100K split of 70,000 training images, 10,000 validation images, and 20,000 test images. Because the labels for the test set are not publicly available, all quantitative results in this paper are reported on the 10K validation set. Before being fed into the network, the input images are resized from the original resolution of 720×1280 to 384×640. The details of data augmentation and the training configuration are presented in the Implementation details subsection.

Unlike BDD100K, TuSimple mainly consists of highway scenes with clearer lane structure and narrower contextual variation. In this study, the official TuSimple training set is not used for training, hyperparameter tuning, or model selection. Instead, from the 2,782 samples of the official test set, we construct a derived pixel-level evaluation set for lane segmentation by converting the row-wise lane annotations into binary masks through a deterministic rasterization procedure. This derived evaluation set is used consistently for all models in the out-of-domain experiments. Therefore, the results on TuSimple reported in this paper should be interpreted as out-of-domain evaluation results under the pixel-level protocol established in this study, rather than as the official metrics of the TuSimple benchmark. This protocol is therefore intended to test cross-domain transfer of binary lane-mask predictions, not to replace or reinterpret the official TuSimple lane-detection benchmark.

An important difference is that TuSimple does not provide pixel-level segmentation ground truth, but stores lane annotations in a row-wise sampling format. Specifically, for each image, the dataset provides a sequence $h_{samples}={\left\{y_{i}\right\}}_{i=\text{1}}^{N}$, that is, a set of fixed sample ordinates along the vertical axis, shared by all lanes in that image. For the 𝒾-th lane, the annotation does not store a 2D polyline directly but stores the sequence of abscissas ${\left\{x_{i}^{\text{𝒾}}\right\}}_{i=\text{1}}^{N}$, each corresponding one-to-one to $y_{i}\in h_{samples}$. Thus, the point set of the lane is reconstructed as $P^{\text{𝒾}}={\left\{\left(x_{i}^{\text{𝒾}},y_{i}\right)\right\}}_{i=\text{1}}^{N}$. Samples with $x_{i}^{\text{𝒾}}<\text{0}$ are treated as missing-label positions and, at the same time, as breakpoints of the lane. Therefore, instead of connecting the entire sequence into a single polyline, each lane is split into consecutive segments $S_{k}^{\text{𝒾}}$ containing only valid points. This processing is intended to avoid generating spurious labels by interpolation across gaps where the original annotation does not confirm the presence of a lane. The overall procedure for converting TuSimple row-wise annotations into segmentation ground truth is illustrated in Fig 4.

**Fig 4:**
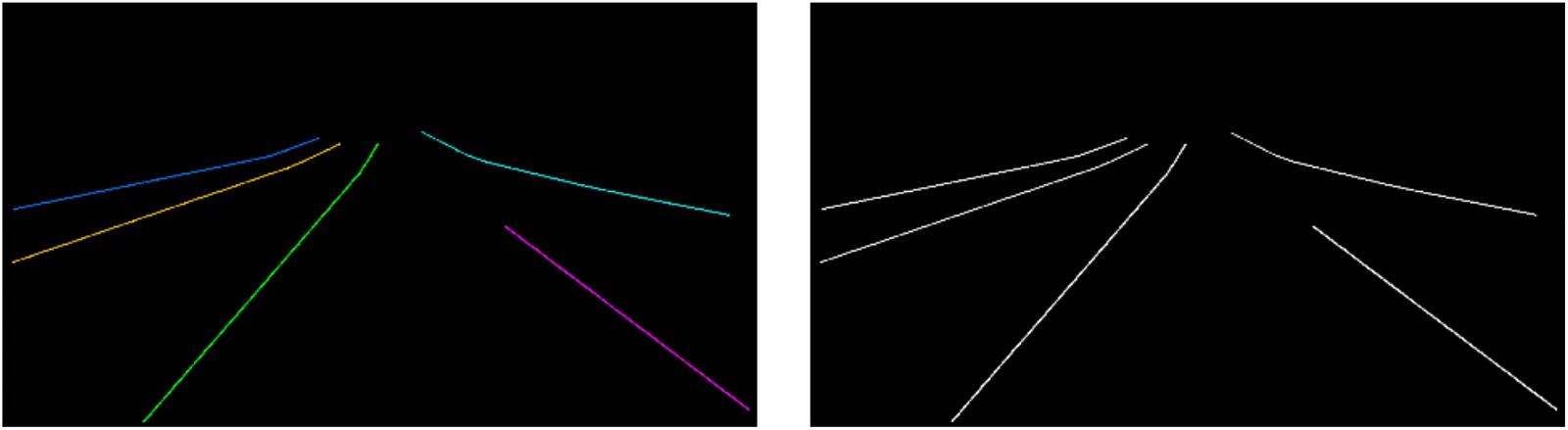
Illustration of the procedure for converting TuSimple row-wise annotations into segmentation ground truth. From left to right: reconstructed 2D polylines and the corresponding rasterized binary mask. The schematic was created by the authors based on the TuSimple annotation format [31] and the deterministic conversion procedure described in this section.

Each segment $S_{k}^{\text{𝒾}}$ is then rasterized onto a binary canvas $M_{\text{0}}$ with the same size as the original image, $\left(H_{\text{0}},W_{\text{0}})=(\text{7}\text{2}\text{0},\text{1}\text{2}\text{8}\text{0}\right)$. Coordinates outside the image boundary are clipped to the valid image domain before drawing. Each valid TuSimple lane segment is rasterized at the original image resolution with a 2-pixel stroke and without anti-aliasing. The 2-pixel width was selected to approximate the relative thinness of the BDD100K lane annotations after downscaling to the common 360 × 640 evaluation resolution. This choice reduces the mask-morphology mismatch introduced when TuSimple row-wise samples are converted into dense binary targets. It therefore supports a more controlled cross-domain transfer test, while the resulting evaluation remains a derived pixel-level protocol rather than the official TuSimple benchmark. Let $R(S_{k}^{\text{𝒾}})$ denote the set of pixels obtained by rasterizing segment $S_{k}^{\text{𝒾}}$. The mask at the original resolution is then defined as


$$\begin{array}{c} M_{\text{0}}(u,v)=\left\{ \begin{array}{cc} \text{2}\text{5}\text{5}, & if\;(u,v)\in {⋃}_{l}{⋃}_{k}R\left(S_{k}^{\text{𝒾}}\right),\\ \text{0}, & otherwise. \end{array}\right. \end{array}$$ (7)

To bring this mask into the evaluation domain, it is resized to 360×640 by bilinear interpolation, denoted $M_{r}=T_{\text{3}\text{6}\text{0}\times \text{6}\text{4}\text{0}}(M_{\text{0}})$, and then binarized again as


$$\begin{array}{c} M(u,v)=1\left[M_{\text{r}}(u,v)>\text{1}\right] \end{array}$$ (8)

Rasterizing at the original resolution and only then downscaling preserves lane geometry better than drawing directly at low resolution, while also making the TuSimple evaluation protocol more compatible with the binary segmentation label format used in this study. To ensure fairness across models, all evaluations on TuSimple in this paper use the same precomputed mask set generated by this deterministic procedure, rather than allowing each method to use its own ground-truth conversion process.

### Evaluation metrics

In this study, the problem is evaluated as binary segmentation with two classes, background and lane. From the final prediction map of the model, we accumulate the numbers of correct and incorrect pixels over the entire evaluation set to obtain the quantities TP, TN, FP, and FN, corresponding to correctly predicted lane pixels, correctly predicted background pixels, background pixels misclassified as lane, and missed lane pixels, respectively.

The central metric of this study is the Intersection over Union (IoU) of the lane class only, denoted ${\text{IoU}}_{lane}$


$$\begin{array}{c} {\text{IoU}}_{lane}=\frac{TP}{TP+FP+FN} \end{array}$$ (9)

According to Eq. (9), ${\text{IoU}}_{\text{lane}}$ directly measures the overlap between the predicted lane region and the reference lane region, while penalizing the two major types of errors in lane segmentation, namely missed lanes and overpredicted lanes. Because the lane class usually occupies only a small fraction of the pixels compared with the background, ${\text{IoU}}_{lane}$ is chosen as the primary metric throughout the experimental section.

In addition, we also report lane recall, denoted${\text{Recall}}_{lane}$


$$\begin{array}{c} {\text{Recall}}_{lane}=\frac{TP}{TP+FN} \end{array}$$ (10)

${\text{Recall}}_{lane}$measures the proportion of lane pixels in the ground truth that are correctly identified as lane by the model, thereby directly reflecting the extent of missed-lane errors. This metric is particularly useful in situations involving thin lanes, partial occlusion, contrast degradation, or discontinuities, where a model may still produce relatively clean predictions yet lose a substantial portion of the lane structure.

To provide an additional perspective on the class imbalance between lane and background, we use balanced accuracy, denoted${\text{Acc}}_{bal}$


$$\begin{array}{c} {\text{Acc}}_{bal}=\frac{\text{1}}{\text{2}}\left(\frac{TP}{TP+FN}+\frac{TN}{TN+FP}\right) \end{array}$$ (11)

From Eq. (11), ${\text{Acc}}_{bal}$ is the average of the sensitivity on the lane class and the specificity on the background class. Therefore, it indicates whether the model maintains a balance between lane detection capability and the ability to limit false positives on the background. Compared with overall pixel accuracy, ${\text{Acc}}_{bal}$ is less dominated by the overwhelming number of background pixels and is therefore more suitable for the class-imbalance characteristics of lane segmentation.

It should be noted that in some previous works on lane segmentation for BDD100K, the quantity TP/(TP+FN) has sometimes been reported under generic names such as accuracy, lane accuracy, or pixel accuracy of lanes. However, such naming can easily be confused with balanced accuracy and overall pixel accuracy. Therefore, in this paper, we consistently use the explicit notation ${\text{Recall}}_{\text{lane}}$ for the quantity TP/(TP+FN). This convention is intended only to standardize the terminology according to the true mathematical nature of the metric and does not alter the meaning of previously published results.

Based on the above considerations, the performance of the model in this paper is characterized primarily by the metric triplet $\left({\text{IoU}}_{lane},\;{\text{Recall}}_{lane},\;{\text{Acc}}_{bal}\right)$. Among them, ${\text{IoU}}_{lane}$ is the main metric for comparing lane segmentation performance, ${\text{Recall}}_{lane}$ is used to analyze the degree of missed-lane error, and ${\text{Acc}}_{bal}$ provides a complementary view of the balance between the lane and background classes.

The absolute value of lane IoU should be interpreted carefully. Lane markings occupy only a very small fraction of image pixels, and the reference masks are thin, so even a small lateral displacement or thickness mismatch can strongly reduce IoU. In the BDD100K lane-mask protocol used in this study, the model is trained with thicker lane targets of 8 pixels to stabilize optimization, whereas validation is performed against thinner 4-pixel lane masks. This train-validation mask-morphology gap makes IoU particularly conservative because the metric penalizes small thickness mismatches and lateral shifts at the pixel level. For this reason, an IoU value in the mid-30% range on BDD100K should not be interpreted in the same way as IoU values for large semantic objects such as road, sky, or vehicles. It is a strict overlap measure for sparse lane pixels. Accordingly, the reported lane IoU demonstrates relative segmentation quality under the BDD100K pixel-level protocol, but it is not by itself sufficient to claim safety readiness for autonomous driving. A safety-critical deployment would require additional validation, including temporal consistency, lane-instance reconstruction, downstream planning impact, failure monitoring, and testing under rare or adverse driving conditions.

### Implementation details

All experiments were conducted using an NVIDIA RTX 5090 GPU and PyTorch 2.9.1. Input images were resized from 720 × 1280–384 × 640 and normalized to [0,1]. HSCM-Lane-S uses a ResNet18 backbone initialized from pretrained weights. The S/M/L variants share the exported feature interface, HSCM placement at 1/4, 1/8, and 1/16, window size 8, depth 2, projected static-sum fusion with α = 0.5, loss function, optimizer, augmentation, and training schedule. For HSCM-Lane-S, the attention-head counts are 4, 8, and 8 at the three levels. The segmentation head produces two-class logits and bilinearly upsamples them to the input resolution.

During training, we use data augmentations including random rotation, translation, scaling, perspective perturbation, horizontal flip, hue-saturation-value (HSV) color jitter, bilateral blur, Gaussian blur, and random crop. The geometric transformations help make the model more robust to viewpoint changes and perspective distortions. The photometric and blur transformations improve adaptability to illumination variation, road-surface reflections, sensor noise, and image-quality degradation, whereas random crop helps the model become more stable under partial occlusion and scene-layout changes.

For optimization, the model is trained for 80 epochs with a batch size of 12. The optimizer is AdamW [32] with an initial learning rate $\eta_{\text{0}}=\text{5}\times {\text{1}\text{0}}^{-\text{4}}$, weight decay ${\text{1}\text{0}}^{-\text{2}}$, $\beta_{\text{1}}=\text{0}.\text{9}$, $\beta_{\text{2}}=\text{0}.\text{9}\text{9}\text{9}$ and $\varepsilon ={\text{1}\text{0}}^{-\text{8}}$. The learning rate is updated using a linear warm-up strategy during the first 5 epochs, followed by polynomial decay with exponent 1.5. Specifically, at epoch e, the learning rate is defined as


$$\begin{array}{c} \eta (e)=\left\{ \begin{array}{cc} \eta_{\text{0}}\left[\rho +(\text{1}-\rho )\frac{e+\text{1}}{E_{w}}\;\right] & \text{0}\leq e<E_{w}\\ \eta_{\text{0}}{\left(\text{1}-\frac{e-E_{w}}{E-E_{w}}\right)}^{p}\; & E_{w}\leq e<E \end{array}\right. \end{array}$$ (12)

where $\eta_{\text{0}}=\text{5}\times {\text{1}\text{0}}^{-\text{4}}$, ρ=0.1, $E_{w}=\text{5}$, E=80, and p=1.5. According to Eq. (12), the learning rate during the 5 warm-up epochs takes the values $\text{0}.\text{2}\text{8}\eta_{\text{0}},\;\text{0}.\text{4}\text{6}\eta_{\text{0}},\;\text{0}.\text{6}\text{4}\eta_{\text{0}},\;\text{0}.\text{8}\text{2}\eta_{\text{0}}$ and $\eta_{\text{0}}$, respectively, and then decreases over the remaining training epochs according to a polynomial schedule with exponent 1.5. In parallel, the model weights are further smoothed using an exponential moving average with a base decay coefficient of 0.9999.

The architecture-level parameters are linked to the controlled ablation studies. The default configuration uses HSCM at all three encoder levels, window size 8, depth 2, projected static-sum fusion with α = 0.5, and the combined Focal-Tversky objective. Window size 8 and depth 2 were retained because they gave the best quality-cost balance among the tested settings. Projected static-sum fusion was retained because it matched the alternative fusion variants in lane IoU while giving the highest measured FPS. The combined loss was selected because it achieved the highest lane IoU among the tested loss configurations. The fixed coefficient α = 0.5 applies symmetric weighting after channel alignment without adding a learned gate.

The loss coefficients defined in the Loss function subsection and the optimization settings reported above were held fixed across the controlled comparisons so that they did not confound the architectural analysis. The principal no-HSCM and S/M/L configurations are reported as five-run mean ± standard deviation. The remaining component comparisons are representative controlled runs under fixed settings, including lane IoU, lane recall, balanced accuracy, parameter count, and FPS where applicable. The attention-head counts are treated as fixed channel-allocation settings and are reported for reproducibility rather than as independently optimized choices.

### Statistical analysis

For the no-HSCM baseline and the HSCM-Lane-S, HSCM-Lane-M, and HSCM-Lane-L configurations, results are reported as the mean ± sample standard deviation over five independent training runs. Approximate two-sided 95% confidence intervals for mean lane IoU were calculated using Student’s t distribution with four degrees of freedom. The remaining component ablations are representative controlled runs and are interpreted descriptively; no formal hypothesis tests were performed.

### Controlled ablation studies

Component ablations use HSCM-Lane-S with the default settings specified in the Implementation details subsection. The controlled study examines the presence of HSCM, encoder placement, window size and depth, decoder fusion, loss composition, and backbone capacity.

HSCM-Lane-S is used as the reference for component ablations for three reasons. First, its ResNet18 backbone matches the no-HSCM counterpart, which isolates the contribution of HSCM without changing backbone capacity. Second, it is the smallest HSCM-Lane configuration, making the complete placement, window, depth, fusion, and loss studies computationally tractable. Third, keeping one compact reference prevents backbone capacity from confounding the component comparisons. HSCM-Lane-M and HSCM-Lane-L are therefore reserved for the separate backbone-capacity analysis.

Five-seed means ± standard deviations are reported for the no-HSCM baseline and the S/M/L variants. Component ablations use representative controlled runs under fixed settings to identify design trends. Accordingly, small differences among the five-seed results, rounded comparative values, and representative component-ablation results should be interpreted within their stated protocols.

Table 3 supports two analyses. Its first two rows isolate the effect of HSCM with ResNet18 fixed, while the three HSCM-Lane rows compare the S/M/L backbones under identical HSCM, decoder, loss, and training settings.

**Table 3:** Five-seed BDD100K results for (i) the HSCM contribution under ResNet18 and (ii) the S/M/L backbone-capacity trade-off.

Configuration	Backbone	n	${𝐈𝐨𝐔}_{lane}$ (%)	${𝐑𝐞𝐜𝐚𝐥𝐥}_{lane}$ (%)	${𝐀𝐜𝐜}_{bal}$ (%)	Params (M)	FPS
No HSCM	ResNet18	5	33.00 ± 0.12	62.48 ± 0.04	80.38 ± 0.04	3.09	259.42 ± 5.58
HSCM-Lane-S	ResNet18	5	34.44 ± 0.13	65.00 ± 0.19	82.12 ± 0.08	5.17	104.04 ± 6.61
HSCM-Lane-M	ResNet34	5	34.86 ± 0.05	66.22 ± 0.11	82.76 ± 0.05	10.56	83.49 ± 8.28
HSCM-Lane-L	ResNet50	5	34.98 ± 0.04	66.66 ± 0.11	82.96 ± 0.05	11.62	70.14 ± 7.66

Note. All HSCM variants use the same HSCM, decoder, loss, and training settings. FPS is measured at 384 × 640 on an NVIDIA RTX 5090 under the implementation described above.

#### Overall effect of HSCM under a fixed ResNet18 backbone.

The first ablation isolates the overall contribution of HSCM. The two configurations use the same ResNet18 backbone, decoder, loss function, training schedule, data augmentation, and evaluation protocol. The only architectural difference is whether the three HSCM stages are inserted into the encoder. This subsection therefore interprets only the first two rows of Table 3. HSCM-Lane-M and HSCM-Lane-L are not discussed here because changing the backbone would confound the isolated effect of HSCM.

The first two rows of Table 3 show that HSCM improves all three main metrics over five random seeds. Compared with the no-HSCM counterpart, HSCM-Lane-S increases lane IoU from 33.00% to 34.44%, lane Recall from 62.48% to 65.00%, and Balanced Accuracy from 80.38% to 82.12%. The largest absolute gain is observed in Recall, suggesting that multi-level shifted-window context modeling mainly helps recover lane pixels that are thin, distant, weakly visible, or fragmented. At the same time, the improvement in IoU and Balanced Accuracy indicates that the higher Recall is not obtained simply by over-expanding the predicted lane mask. The repeated-run results indicate that the observed improvement is consistent under the training protocol used in this study. Based on the five-run means and standard deviations, approximate 95% confidence intervals for lane IoU are 33.00 ± 0.15% for the no-HSCM baseline and 34.44 ± 0.16% for HSCM-Lane-S. These intervals are reported as descriptive uncertainty estimates for the five-run means rather than as a substitute for a formal statistical hypothesis test.

#### Placement of HSCM in the encoder.

The next ablation evaluates where HSCM should be inserted in the encoder. The candidate levels correspond to the 1/4, 1/8, and 1/16 feature resolutions, denoted as C1, C2, and C3, respectively. All variants use the same backbone, decoder, loss function, window size, HSCM depth, and training setting. This experiment therefore isolates the effect of using HSCM at shallow, intermediate, deep, or multi-level encoder stages.

All single-level HSCM placements improve over the no-HSCM reference, indicating that shifted-window context modeling is useful even when applied at only one encoder level. However, the best representative result is obtained when HSCM is used jointly at the 1/4, 1/8, and 1/16 levels. This pattern is consistent with the intended multi-level design: the 1/4 level retains relatively fine lane geometry, the 1/8 level provides an intermediate balance between detail and context, and the 1/16 level contributes broader semantic road-layout information. Although the margins among the multi-level variants are modest, the full three-level placement gives the highest IoU, Recall, and Balanced Accuracy among the tested settings. It is therefore retained as the default configuration. The corresponding results are summarized in Table 4.

**Table 4:** HSCM placement ablation.

Setting	HSCM	${𝐈𝐨𝐔}_{lane}$ (%)	${𝐑𝐞𝐜𝐚𝐥𝐥}_{lane}$ (%)	${𝐀𝐜𝐜}_{bal}$ (%)	Params (M)	FPS
No HSCM	–	33.2	62.5	80.4	3.09	258.83
$C_{\text{1}}$	1/4	34.2	64.1	81.7	3.19	179.09
$C_{\text{2}}$	1/8	34.2	64.2	81.7	3.49	174.74
$C_{\text{3}}$	1/16	34.1	63.9	81.6	4.67	145.46
$C_{\text{1}}+C_{\text{2}}$	1/4 + 1/8	34.4	64.6	81.9	3.59	138.65
$C_{\text{1}}+C_{\text{3}}$	1/4 + 1/16	34.3	64.7	82.0	4.77	142.44
$C_{\text{2}}+C_{\text{3}}$	1/8 + 1/16	34.3	64.7	82.0	5.07	138.51
$C_{\text{1}}+C_{\text{2}}+C_{\text{3}}$	1/4 + 1/8 + 1/16	34.5	65.0	82.1	5.17	111.22

Note. $C_{\text{1}}$, $C_{\text{2}}$, and $C_{\text{3}}$ denote the 1/4, 1/8, and 1/16 encoder levels; all other settings are fixed.

#### Window size and HSCM depth.

This ablation studies two HSCM hyperparameters: the attention window size and the number of Swin-style blocks per HSCM stage. The default configuration uses window size 8 and depth 2. All other architectural and training settings are fixed.

The tested window sizes were selected to keep the ablation controlled across all HSCM feature levels. For the 384 × 640 input, HSCM operates on 96 × 160, 48 × 80, and 24 × 40 feature maps. Window sizes 4 and 8 divide all three resolutions without requiring padding, cropping, or non-uniform window masking. They also represent two practical regimes: a more local 4 × 4 context and a broader 8 × 8 context. Larger windows would either require additional padding/masking at the 1/16 feature level or make the deepest-stage attention less comparable, thereby introducing another implementation variable. Similarly, depths 2, 4, and 6 correspond to one, two, and three W-MSA/SW-MSA block pairs. Depth 2 is the minimal shifted-window configuration, while depths 4 and 6 test whether additional context-modeling capacity brings further gains. The tested configurations are summarized in Table 5.

**Table 5:** Ablation of HSCM window size and depth on HSCM-Lane-S.

Window size	Depth	${𝐈𝐨𝐔}_{lane}$ (%)	${𝐑𝐞𝐜𝐚𝐥𝐥}_{lane}$ (%)	${𝐀𝐜𝐜}_{bal}$ (%)	Params (M)	FPS
4	2	34.3	64.7	82.0	5.17	107.86
8	2	34.5	65.0	82.1	5.17	111.22
8	4	34.5	65.0	82.1	7.24	72.31
8	6	34.5	65.1	82.2	9.32	53.68

Note. All other settings are fixed.

Window size 8 provides slightly higher IoU, Recall, and Balanced Accuracy than window size 4 in the representative run. It is also marginally faster in this implementation, likely because the larger window size reduces the number of local windows processed at each feature level. Increasing the HSCM depth from 2 to 4 or 6 does not improve lane IoU, while it substantially increases the parameter count and reduces inference speed. Depth 6 gives only a marginal Recall and Balanced Accuracy increase, but this gain is not reflected in IoU and comes with a large speed penalty. Therefore, window size 8 and depth 2 are selected as the default HSCM setting because they provide the best quality-cost balance among the tested variants.

#### Decoder fusion strategy.

To isolate the contribution of the decoder fusion strategy, we compare the proposed projected static-sum fusion with concatenation-based fusion and gated fusion. All variants use the same HSCM-Lane-S encoder configuration, HSCM placement, window size, HSCM depth, loss function, and training schedule. The corresponding results are summarized in Table 6.

**Table 6:** Ablation of decoder fusion on HSCM-Lane-S.

Fusion strategy	${𝐈𝐨𝐔}_{lane}$ (%)	${𝐑𝐞𝐜𝐚𝐥𝐥}_{lane}$ (%)	${𝐀𝐜𝐜}_{bal}$ (%)	Params (M, rounded)	FPS
Projected static-sum	34.5	65.0	82.1	5.17	111.22
Concatenation	34.5	65.4	82.3	5.17 (+ < 0.01)	104.14
Gated fusion	34.5	65.0	82.1	5.17 (+ < 0.01)	101.98

Note. HSCM, loss, and training settings are fixed.

Parameter counts are rounded to two decimal places. Concatenation and gated fusion introduce only a small number of additional decoder parameters, below 0.01M, so all three fusion variants round to 5.17M.

All three fusion strategies obtain the same lane IoU in the representative run. Concatenation slightly increases Recall and Balanced Accuracy, but this does not translate into a higher IoU and is accompanied by lower inference speed. Gated fusion also does not improve IoU and is slower than projected static-sum fusion. These results suggest that a more complex fusion mechanism is not necessary in the decoder when multi-level context modeling has already been introduced in the encoder. Projected static-sum fusion is therefore retained as the default decoder design because it achieves the same overlap quality as the alternatives while providing the highest inference speed among the tested fusion variants.

#### Loss components.

We further ablate the loss function to evaluate the respective roles of the Focal and Tversky components. The same HSCM-Lane-S architecture is trained with Focal loss only, Tversky loss only, and the combined Focal-Tversky objective. The corresponding results are summarized in Table 7.

**Table 7:** Ablation of loss components on HSCM-Lane-S.

Loss	${𝐈𝐨𝐔}_{lane}$ (%)	${𝐑𝐞𝐜𝐚𝐥𝐥}_{lane}$ (%)	${𝐀𝐜𝐜}_{bal}$ (%)
Focal only	32.0	74.5	86.7
Tversky only	33.7	60.4	79.9
Focal + Tversky	34.5	65.0	82.1

Note. Architecture and training settings are fixed.

Focal loss alone produces the highest Recall and Balanced Accuracy, but its lane IoU decreases to 32.0%. This indicates that emphasizing difficult pixels can increase lane-pixel recovery but does not by itself provide the best pixel-level overlap with the reference mask. Tversky loss alone gives a more conservative prediction pattern, with lower Recall and lower Balanced Accuracy. The combined Focal-Tversky objective achieves the highest IoU among the tested losses, suggesting that the two terms play complementary roles: the Focal component helps emphasize difficult sparse lane pixels, whereas the Tversky component helps regulate overlap quality under severe lane/background imbalance. The combined objective is therefore used as the default training loss.

#### ResNet backbone capacity Under the fixed HSCM-Lane design.

Returning to the three HSCM-Lane rows of Table 3, this subsection evaluates the effect of ResNet backbone capacity. The three experimental configurations follow the same HSCM-Lane design and differ only in the ResNet backbone: HSCM-Lane-S uses ResNet18, HSCM-Lane-M uses ResNet34, and HSCM-Lane-L uses ResNet50. All three configurations use HSCM at the 1/4, 1/8, and 1/16 levels, window size 8, HSCM depth 2, projected static-sum fusion, and the Focal-Tversky objective.

Backbone scaling improves segmentation quality, but the gains gradually saturate. Compared with HSCM-Lane-S, HSCM-Lane-M improves lane IoU by 0.42 points and lane Recall by 1.22 points, while reducing speed from 104.04 FPS to 83.49 FPS. HSCM-Lane-L achieves the best segmentation quality, with 34.98% IoU and 66.66% Recall, but its improvement over HSCM-Lane-M is only 0.12 IoU points and 0.44 Recall points while further reducing speed to 70.14 FPS. Therefore, HSCM-Lane-L is preferable when segmentation quality is the main priority, HSCM-Lane-M provides a balanced quality-speed operating point, and HSCM-Lane-S remains the most compact real-time configuration among the evaluated backbone-capacity configurations.

#### Summary of ablation findings.

The ablation study supports the selected HSCM-Lane configuration. First, HSCM provides a stable five-seed improvement over the no-HSCM baseline. Second, using HSCM at all three encoder levels gives the best representative result among the tested placement settings, supporting the use of multi-level context modeling. Third, window size 8 and depth 2 provide the best quality-cost balance, whereas deeper HSCM stages increase computational cost without improving IoU. Fourth, projected static-sum fusion is sufficient for the decoder because concatenation and gated fusion do not improve IoU and reduce inference speed. Fifth, the combined Focal-Tversky objective achieves the best IoU among the tested loss configurations. Finally, higher-capacity ResNet backbones improve quality but with diminishing returns and higher computational cost. These findings justify reporting HSCM-Lane-S/M/L as three backbone-capacity operating points of the same HSCM-Lane design rather than as unrelated architectures.

### Comparative results on BDD100K

Two comparison settings are used. A single-task model is optimized for lane-mask segmentation without jointly predicting additional perception tasks, whereas a multitask model shares features across lane segmentation and one or more additional perception tasks; a lane-line branch denotes only the lane output of such a multitask system. The labels “LL-Seg only” and “segll” follow the cited sources and denote lane-line-segmentation-only configurations. Because published studies do not consistently report lane-class IoU, lane recall, and balanced accuracy together, lane-class IoU is the primary comparison metric, and the other metrics are discussed only when available. The BDD100K comparison tables use published values reported in the cited sources, whereas the TuSimple comparison reports models rerun under the derived pixel-level protocol. HSCM-Lane values in the BDD100K comparisons are five-seed means rounded to one decimal place; their standard deviations are reported in the five-seed analysis above.

#### Comparison with single-task models.

As shown in Table 8, HSCM-Lane-S achieves 34.4% lane IoU, matching TwinLiteNet+ (Lane only) among the single-task models considered. HSCM-Lane-M and HSCM-Lane-L further reach 34.9% and 35.0% lane IoU, respectively, when the backbone is scaled while the HSCM placement, window size, depth, decoder fusion, and loss settings are kept fixed. These results place the proposed model in the competitive group on BDD100K. Compared with early baselines such as ENet, SCNN, and ENet-SAD, HSCM-Lane-S improves lane IoU by 19.8, 18.6, and 18.4 points, respectively. Compared with baselines that are closer in architecture or training setting, the HSCM-Lane-S exceeds YOLOv8 (segll), YOLOP (LL-Seg only), and YOLO-L by 11.5, 6.5, and 6.3 ${\text{IoU}}_{lane}$ points, respectively. These gaps indicate stronger lane-mask overlap under the reported BDD100K protocol, while the comparison remains limited by the fact that the external results are drawn from the cited literature rather than rerun in a single pipeline.

**Table 8:** Comparison with single-task models on BDD100K.

Model	${𝐈𝐨𝐔}_{lane}$ (%)	${𝐑𝐞𝐜𝐚𝐥𝐥}_{lane}\;(\%)$	${𝐀𝐜𝐜}_{bal}$ (%)
ENet [2,12,33]	14.6	34.1	–
SCNN [1,5,12]	15.8	35.8	–
ENet-SAD [2,12]	16.0	36.6	–
YOLOv8 (segll) [34,35]	22.9	–	80.5
YOLOP (LL-Seg only) [5]	27.9	79.6	–
YOLO-L [36,37]	28.1	–	–
TwinLiteNet+ (Lane only) [7]	34.4	–	81.9
HSCM-Lane-S	34.4	65.0	82.1
HSCM-Lane-M	34.9	66.2	82.8
HSCM-Lane-L	35.0	66.7	83.0

Note. Results of previously published methods are taken from the cited references. HSCM-Lane-S/M/L values are evaluated in this work and shown as five-seed means rounded to one decimal place. The corresponding standard deviations are reported in Table 3.

For the supplementary metrics, HSCM-Lane-S reaches ${\text{Acc}}_{bal}=\text{8}\text{2}.\text{1}\%$, which is higher than YOLOv8 (segll) at 80.5% and slightly higher than TwinLiteNet+ (Lane only) at 81.9%. Notably, YOLOP (LL-Seg only) has a higher ${\text{Recall}}_{lane}$, reaching 79.6%, but its ${\text{IoU}}_{lane}$ is only 27.9%. This indicates that a high recall rate alone is not sufficient to guarantee good overlap quality at the pixel level. According to the central metric ${\text{IoU}}_{lane}$, HSCM-Lane achieves a better balance between preserving lane structure and controlling overprediction. Overall, Table 8 indicates that HSCM-Lane belongs to the competitive group among the strongest reported single-task lane-segmentation baselines under the BDD100K pixel-level protocol.

#### Comparison with related multitask perception models on the lane-line segmentation branch.

In the broader setting that includes lane-line segmentation branches of multitask perception systems and several strong baselines, Table 9 shows that HSCM-Lane-S achieves 34.4% lane IoU, slightly above TwinLiteNet+ (multi-task), while HSCM-Lane-M and HSCM-Lane-L reach 34.9% and 35.0%, respectively. HSCM-Lane-L is numerically highest in this comparison table, but the differences among the strongest methods remain narrow. The compact HSCM-Lane-S configuration is therefore interpreted as competitive with the strongest multitask reference. HSCM-Lane-S is numerically above TwinLiteNet+ (multi-task) by 0.2 points, IALaneNet by 1.9 points, TwinMixing by 2.1 points, SegFormer by 2.7 points, HybridNets by 2.8 points, Sparse U-PDP (w/o Detection) by 3.2 points, TwinLiteNet by 3.3 points, DeepLabV3+ by 4.6 points, YOLOP (multi-task) by 8.2 points, and YOLOv8 (multi-task) by 10.1 points. The margin of only 0.2 points over TwinLiteNet+ indicates that the strongest competing configuration has come very close, so the HSCM-Lane-S result should be interpreted as a modest IoU advantage rather than a large separation. HSCM-Lane-M and HSCM-Lane-L further show that increasing backbone capacity under the same HSCM placement, window size, depth, decoder fusion, and loss settings yields gradual additional gains.

**Table 9:** Comparison with related multitask models on the lane-line segmentation branch of BDD100K.

Model	${𝐈𝐨𝐔}_{lane}$ (%)	${𝐑𝐞𝐜𝐚𝐥𝐥}_{lane}\;(\%)$	${𝐀𝐜𝐜}_{bal}$ (%)
YOLOv8 (multi-task) [34,35]	24.3	–	81.7
YOLOP (multi-task) [5]	26.2	70.5	–
DeepLabV3+ [7,8,20]	29.8	–	–
TwinLiteNet [6,7]	31.1	–	77.8
Sparse U-PDP (w/o Detection) [20]	31.2	–	–
HybridNets [16]	31.6	–	85.4
SegFormer [7,9,20]	31.7	–	–
TwinMixing [25]	32.3	–	–
IALaneNet (ConvNeXt-small) [12]	32.5	–	–
TwinLiteNet+ (multi-task) [7]	34.2	–	81.9
HSCM-Lane-S	34.4	65.0	82.1
HSCM-Lane-M	34.9	66.2	82.8
HSCM-Lane-L	35.0	66.7	83.0

Considering the supplementary metrics, HSCM-Lane-S reaches ${\text{Acc}}_{bal}=\text{8}\text{2}.\text{1}\%$, whereas HSCM-Lane-M and HSCM-Lane-L reach 82.8% and 83.0%, respectively. These values are higher than TwinLiteNet+ at 81.9%, YOLOv8 (multi-task) at 81.7%, and TwinLiteNet at 77.8%, although all three HSCM-Lane variants remain lower than HybridNets at 85.4%. This indicates that the proposed HSCM-Lane configurations improve lane-mask overlap while maintaining competitive lane/background balance, but it should not be described as superior on every reported metric.

At the same time, YOLOP (multi-task) has ${\text{Recall}}_{lane}=\text{7}\text{0}.\text{5}\%$, higher than HSCM-Lane-S, HSCM-Lane-M, and HSCM-Lane-L, which reach 65.0%, 66.2%, and 66.7%, respectively, but its ${\text{IoU}}_{lane}$ reaches only 26.2%. This result once again shows that high recall alone does not fully reflect segmentation quality: a model may retain more lane pixels yet still be inferior in overall overlap if the prediction is not sufficiently compact or does not follow the geometry of the lane mask closely enough. Therefore, in terms of ${\text{IoU}}_{lane}$, HSCM-Lane shows that a specialized single-task architecture, combining a ResNet-based encoder with hierarchical context modeling, remains competitive with the listed multitask lane-line branches in mask-overlap quality, while the small margins near TwinLiteNet+ should be interpreted cautiously.

Taken together, Tables 8 and 9 provide the published-reference accuracy comparison, while Table 3 reports controlled parameter and throughput measurements for the no-HSCM and S/M/L configurations at an input size of 384 × 640 under the reported RTX 5090 environment. The out-of-domain evaluation below complements this evidence with accuracy and FPS for models rerun under the common TuSimple pixel-level protocol. HSCM-Lane offers explicit multi-level context modeling within a simple single-task encoder-decoder and provides three quality-speed operating points. Its main cost is the increase from 3.09M to 5.17M parameters and the reduction from 259.42 to 104.04 FPS when HSCM is added to ResNet18. Computational values from published external baselines are not merged into a single ranking when input resolution, hardware, and timing procedures differ.

### Cross-dataset evaluation on TuSimple under a derived pixel-level protocol

As described in the Datasets and evaluation protocol subsection, TuSimple is used in this study only as an out-of-domain test set. Specifically, from the 2,782 images of the official test set, we construct pixel-level binary masks using a deterministic rasterization procedure and use them consistently for all models. The baselines in Table 10 were all rerun by us from publicly available source code, trained on BDD100K, and evaluated only on TuSimple, without retraining or fine-tuning on this dataset. Therefore, the numbers in Table 10 should be interpreted as a comparison of out-of-domain generalization under the same evaluation protocol, rather than as the official metrics of the TuSimple benchmark.

**Table 10:** Out-of-domain comparison on TuSimple under the pixel-level evaluation protocol of this study.

Model	${𝐈𝐨𝐔}_{lane}$ (%)	${𝐑𝐞𝐜𝐚𝐥𝐥}_{lane}\;(\%)$	${𝐀𝐜𝐜}_{bal}$ (%)	FPS
YOLOP	21.7	69.9	83.8	58.43
HybridNets	25.2	75.3	86.6	14.71
TwinLiteNet+	27.0	63.5	81.0	81.95
HSCM-Lane-S	27.2	67.0	82.7	110.83
HSCM-Lane-M	27.6	66.9	82.7	95.38
HSCM-Lane-L	27.6	67.0	82.7	68.15

According to Table 10, HSCM-Lane-S achieves 27.2% lane IoU, whereas HSCM-Lane-M and HSCM-Lane-L both achieve 27.6%. Thus, all three proposed variants are numerically above TwinLiteNet+ in IoU under the derived pixel-level TuSimple protocol, with HSCM-Lane-M and HSCM-Lane-L obtaining the highest IoU values in this table. However, these margins should be interpreted only within the rasterized-mask protocol of this study and should not be treated as official TuSimple benchmark superiority. Compared with TwinLiteNet + , HSCM-Lane-S has higher ${\text{Recall}}_{lane}$ (67.0% vs. 63.5%), higher ${\text{Acc}}_{bal}$ (82.7% vs. 81.0%), and markedly higher inference speed (110.83 FPS vs. 81.95 FPS). HSCM-Lane-M further increases ${\text{IoU}}_{lane}$ to 27.6% while still running faster than TwinLiteNet+ (95.38 FPS vs. 81.95 FPS). HSCM-Lane-L obtains the same ${\text{IoU}}_{lane}$ as HSCM-Lane-M but is slower than TwinLiteNet+ (68.15 FPS vs. 81.95 FPS), so its advantage on TuSimple is mainly overlap quality rather than speed.

Compared with HybridNets and YOLOP, HSCM-Lane-S improves ${\text{IoU}}_{lane}$ by 2.0 and 5.5 points, respectively, whereas HSCM-Lane-M and HSCM-Lane-L improve ${\text{IoU}}_{lane}$ by 2.4 and 5.9 points, respectively. Although HybridNets and YOLOP have higher ${\text{Recall}}_{lane}$ and ${\text{Acc}}_{bal}$, their lower ${\text{IoU}}_{lane}$ indicates that the overall overlap between the predicted lane mask and the reference mask is still inferior under this protocol. This suggests that higher lane-pixel recovery does not necessarily yield tighter overlap with the thin rasterized lane masks. Taking lane IoU as the primary metric, HSCM-Lane belongs to the competitive group on TuSimple under this derived evaluation protocol, with HSCM-Lane-M/L achieving the highest IoU in Table 10 and HSCM-Lane-S offering the strongest quality-speed trade-off among the HSCM-Lane configurations.

Overall, this result shows that the proposed model retains reasonable cross-dataset transfer capability when transferred from the BDD100K training domain to the highway domain of TuSimple without requiring adaptation on TuSimple. However, because the TuSimple masks are derived from row-wise annotations, these results should be interpreted only within the pixel-level protocol of this study and not as official TuSimple benchmark scores.

### Stability analysis under different scene conditions

To analyze the stability of the model, we further decompose the BDD100K validation set according to three criteria: weather, time of day, and street-scene context. It should be noted that the group sizes in the condition-wise analyses are not uniform. Therefore, strong quantitative conclusions are drawn mainly from groups with a large number of samples, whereas very small groups such as foggy, parking lot, tunnel, gas stations, and some undefined labels are only indicative.

According to Table 11, HSCM-Lane maintains good performance under common weather conditions. Among the groups with sufficiently large sample size, overcast yields the highest results, with 36.0% ${\text{IoU}}_{lane}$, 70.1% ${\text{Recall}}_{lane}$, and 84.6% ${\text{Acc}}_{bal}$. The undefined group also attains high values of 35.7%, 69.1%, and 84.2%, while partly cloudy reaches 35.5%, 67.7%, and 83.4%, and clear reaches 34.0%, 63.8%, and 81.5%. In contrast, performance drops markedly under snowy and rainy conditions, where ${\text{IoU}}_{lane}$ is 32.1% for both, and ${\text{Recall}}_{lane}$ is 57.8% and 57.9%, respectively. The foggy group yields even lower results, but it contains only 13 images and is therefore mainly indicative. This trend suggests that adverse weather conditions primarily increase missed-lane errors. In fog, rain, and snow, the main difficulty is that thin lane markings lose contrast against the road surface, especially in the far field, so the model tends to produce false negatives on weak or partially invisible lane pixels rather than only making boundary-placement errors.

**Table 11:** Performance under different weather conditions on BDD100K.

Condition	Images	${𝐈𝐨𝐔}_{lane}$ (%)	${𝐑𝐞𝐜𝐚𝐥𝐥}_{lane}\;(\%)$	${𝐀𝐜𝐜}_{bal}$ (%)
Clear	5,346	34.0	63.8	81.5
Overcast	1,239	36.0	70.1	84.6
Undefined	1,157	35.7	69.1	84.2
Snowy	769	32.1	57.8	78.7
Partly cloudy	738	35.5	67.7	83.4
Rainy	738	32.1	57.9	78.7
Foggy	13	29.3	50.2	74.8

The results by time of day in Table 12 show that the model performs best during daytime, with 35.4% ${\text{IoU}}_{lane}$, 67.4% ${\text{Recall}}_{lane}$, and 83.3% ${\text{Acc}}_{bal}$. Performance decreases slightly at dawn/dusk to 34.2%, 64.6%, and 81.9%, and drops more clearly at night to 33.2%, 61.6%, and 80.4%. The undefined group reaches only 27.0%, 43.4%, and 71.5%, but it contains only 35 images and is therefore mainly indicative. This shows that weak illumination and low contrast remain substantial difficulties for lane segmentation. At night, this degradation is further amplified by headlight glare, road-surface reflections, and reduced color contrast, which make thin lane markings visually similar to background artifacts and therefore increase both missed detections and local false positives.

**Table 12:** Performance by time of day on BDD100K.

Condition	Images	${𝐈𝐨𝐔}_{lane}$ (%)	${𝐑𝐞𝐜𝐚𝐥𝐥}_{lane}\;(\%)$	${𝐀𝐜𝐜}_{bal}$ (%)
Daytime	5,258	35.4	67.4	83.3
Night	3,929	33.2	61.6	80.4
Dawn/Dusk	778	34.2	64.6	81.9
Undefined	35	27.0	43.4	71.5

According to Table 13, HSCM-Lane is fairly stable across the three most frequent contexts, namely city street, residential, and highway, with ${\text{IoU}}_{lane}$ values of 34.7%, 34.7%, and 33.9%, respectively. Among them, residential yields the highest ${\text{Recall}}_{lane}$ and ${\text{Acc}}_{bal}$, reaching 66.6% and 83.1%, whereas city street ties for the highest ${\text{IoU}}_{lane}$ at 34.7%. The undefined group reaches 31.5%, 55.3%, and 77.5%, but contains only 53 images and is therefore mainly indicative. In contrast, performance drops substantially in parking lot (24.4%, $IoU_{lane}$), tunnel (27.0%), and gas stations (18.3%). However, these groups have very small sample sizes and thus mainly indicate a tendency for the model to still encounter difficulty in rare scenes or scenes with nonstandard lane structure. The likely reason is that these scenes differ from the dominant city-street, residential, and highway patterns in both geometry and appearance. Parking lots often contain short, crossing, or fragmented markings that do not follow a single road-perspective layout. Tunnels introduce abrupt illumination changes, shadows, and reflective surfaces. Gas-station scenes contain lane-like pavement markings, curbs, signs, and artificial lighting that can be confused with true lane markings. These factors weaken the continuity prior learned from common road scenes and explain why the errors in these groups are dominated by missed thin lane pixels and occasional confusion with lane-like background structures.

**Table 13:** Performance by road-scene category on BDD100K.

Condition	Images	${𝐈𝐨𝐔}_{lane}$ (%)	${𝐑𝐞𝐜𝐚𝐥𝐥}_{lane}\;(\%)$	${𝐀𝐜𝐜}_{bal}$ (%)
City street	6,112	34.7	66.0	82.6
Highway	2,499	33.9	62.5	80.8
Residential	1,253	34.7	66.6	83.1
Undefined	53	31.5	55.3	77.5
Parking lot	49	24.4	37.2	68.5
Tunnel	27	27.0	43.2	71.4
Gas stations	7	18.3	28.0	64.0

Taken together, Tables 11–13 show that HSCM-Lane is stable under common BDD100K conditions but remains sensitive to adverse weather, unfavorable illumination, and rare contexts. These condition-wise results identify the main failure modes and motivate future work on robustness to adverse illumination, rare scene layouts, and ambiguous lane-like background structures. The Qualitative results subsection provides complementary qualitative comparisons of the isolated HSCM effect and out-of-domain behavior.

### Qualitative results

This subsection provides qualitative evidence that complements the quantitative evaluation reported above. The analysis comprises two comparisons: the no-HSCM baseline versus HSCM-Lane on BDD100K and out-of-domain behavior on TuSimple. The panels show annotation-derived ground-truth masks, model predictions, and error maps. In the error maps, cyan denotes true-positive lane pixels, yellow denotes false-positive lane pixels, and red denotes false-negative lane pixels. A desirable prediction should therefore contain continuous cyan lane structures while keeping yellow and red regions local and limited.

#### Direct qualitative comparison between no-HSCM and HSCM-Lane.

To isolate the visual effect of HSCM, Fig 5 compares the no-HSCM baseline and HSCM-Lane on the same BDD100K scene under the same visualization layout. In this comparison, the no-HSCM baseline can recover the main lane layout, but several predicted lane segments remain short and fragmented, especially in distant or weakly visible regions. This behavior is reflected in the error map as red regions along the annotated lanes. By contrast, HSCM-Lane produces more continuous cyan bands and fewer red gaps, while the yellow regions remain localized. This visual pattern is consistent with the five-seed ablation results: HSCM improves lane IoU, lane Recall, and Balanced Accuracy, and the higher Recall is not obtained by simply over-expanding the predicted lane mask.

**Fig 5:**
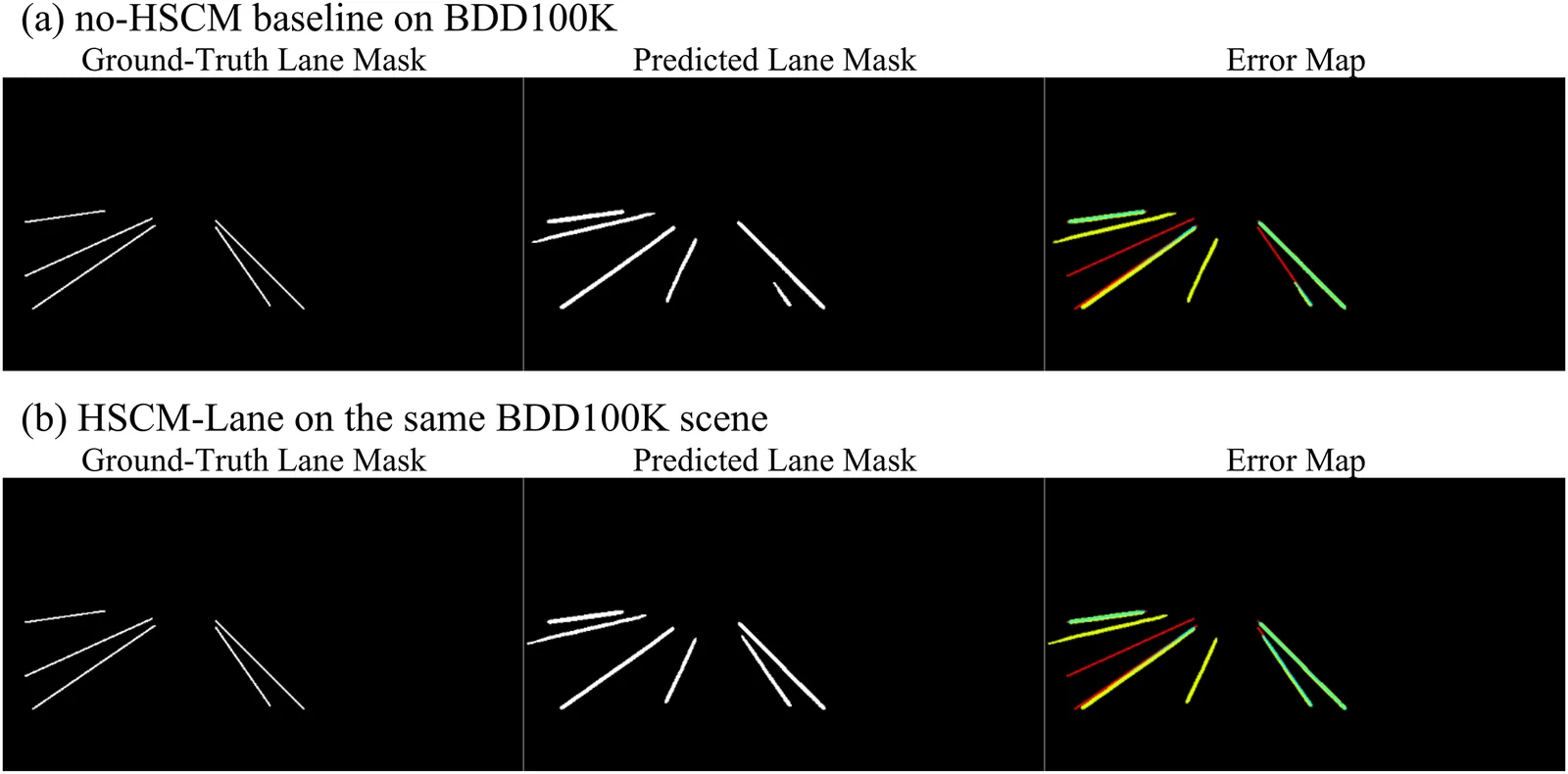
Direct qualitative comparison on BDD100K between (a) the no-HSCM baseline and (b) HSCM-Lane. Each panel shows ground-truth lane mask, predicted lane mask, and error map. The visualizations were generated by the authors from BDD100K lane annotations [15] and model outputs.

#### Out-of-domain qualitative results on TuSimple.

Fig 6 extends the qualitative comparison to TuSimple to illustrate the out-of-domain behavior under the derived pixel-level protocol used in this paper. Under the derived pixel-level protocol used in this study, YOLOP can identify the main highway lane layout, but its prediction is more fragmented in several long lane regions. HSCM-Lane produces longer and more continuous lane structures, especially along lanes extending toward the far field. The error map contains more continuous cyan bands and fewer red gaps on the main lane markings, without a substantial expansion of yellow false-positive regions.

**Fig 6:**
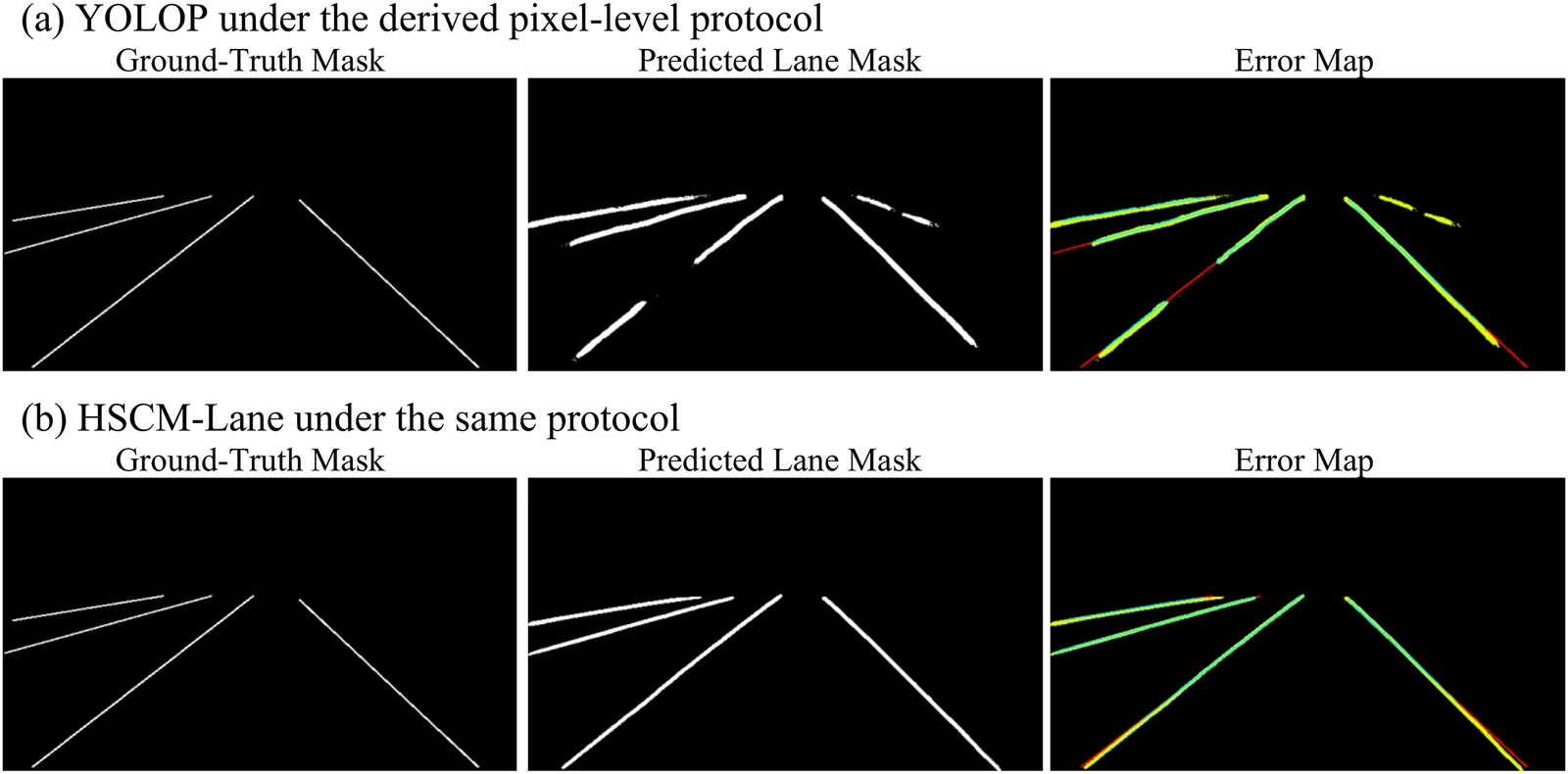
Out-of-domain comparison on TuSimple under the derived pixel-level protocol. (a) YOLOP and (b) HSCM-Lane. The visualizations were generated by the authors from TuSimple lane annotations [31] and model outputs.

These TuSimple examples should be interpreted only within the derived pixel-level out-of-domain protocol of this paper, not as official TuSimple benchmark results. Qualitatively, they are consistent with the quantitative out-of-domain results: HSCM-Lane transfers reasonably to highway scenes without target-domain fine-tuning, but errors remain in distant or very thin lane regions.

Overall, the two qualitative comparisons are consistent with the quantitative analysis. In the selected BDD100K example, HSCM-Lane produces more continuous lane predictions and fewer missed lane pixels than the no-HSCM counterpart. The TuSimple comparison illustrates cross-domain transfer without target-domain fine-tuning, whereas the condition-wise results reported above show continuing sensitivity to adverse illumination, weather degradation, rare layouts, and lane-like background structures. Therefore, the qualitative evidence should be read as explanatory support for the measured trends, not as a substitute for broader safety-oriented validation.

## Discussion

This section interprets the experimental evidence rather than repeating the full results. The main question is whether the proposed context modeling provides a useful quality gain under a realistic computational budget, how the absolute lane-IoU values should be interpreted, and what limitations remain before practical deployment.

### Cost-benefit trade-off and interpretation of the main results

The present findings should be interpreted within several related lines of research. SCNN [1] established the value of explicit spatial information propagation for elongated lane structures, while ENet-SAD [2] showed that attention-derived contextual supervision can strengthen lightweight lane models. Swin Transformer [10] introduced shifted-window attention as an efficient mechanism for cross-window interaction. HSCM-Lane adapts that principle to intermediate ResNet features rather than replacing the convolutional backbone. In parallel, YOLOP [5] and TwinLiteNet+ [7] illustrate the system-level value of shared multitask perception. HSCM-Lane instead isolates the lane-mask objective so that the effect of multi-level context modeling can be examined without cross-task coupling.

The experimental results show that this design direction is effective. Over five seeds on BDD100K, HSCM-Lane-S improves lane IoU from 33.00 ± 0.12% to 34.44 ± 0.13%, lane Recall from 62.48 ± 0.04% to 65.00 ± 0.19%, and Balanced Accuracy from 80.38 ± 0.04% to 82.12 ± 0.08% compared with the no-HSCM counterpart. The largest gain is in Recall, which indicates that multi-level shifted-window context mainly helps recover thin, distant, weakly visible, or fragmented lane pixels. The simultaneous gain in IoU and Balanced Accuracy shows that this improvement is not obtained simply by over-expanding the predicted lane mask.

Expressed as effect magnitudes, these changes equal absolute gains of 1.44 percentage points in lane IoU, 2.52 points in lane Recall, and 1.74 points in Balanced Accuracy. Relative to the no-HSCM means, the gains are 4.36%, 4.03%, and 2.16%, respectively. The approximate 95% confidence intervals for the five-run mean lane IoU are 33.00 ± 0.15% for the no-HSCM model and 34.44 ± 0.16% for HSCM-Lane-S. These intervals provide descriptive uncertainty estimates for the repeated-run results and should not be interpreted as a formal test of statistical significance.

External comparisons require a conservative interpretation. HSCM-Lane-S matches TwinLiteNet+ (Lane only) at 34.4% lane IoU and is 0.2 percentage points above TwinLiteNet+ (multi-task), while HSCM-Lane-L reaches 35.0%, only 0.6 points above the lane-only reference. These margins support the conclusion that HSCM-Lane is competitive rather than substantially superior. Its main structural distinction is the use of a dedicated single-task ResNet encoder with multi-level shifted-window context, whereas multitask systems share representations across lane segmentation and additional perception tasks.

The benefit is not cost-free. Adding HSCM increases the parameter count from 3.09M to 5.17M and reduces the measured speed from 259.42 FPS to 104.04 FPS. However, HSCM-Lane-S remains real-time in the reported RTX 5090 setting, and the improvement is stable across repeated runs. Backbone scaling provides additional quality but with diminishing returns: HSCM-Lane-M reaches 34.86 ± 0.05% IoU at 83.49 FPS, whereas HSCM-Lane-L reaches 34.98 ± 0.04% IoU at 70.14 FPS. Thus, HSCM-Lane-S is the compact operating point, HSCM-Lane-M is a balanced quality-speed option, and HSCM-Lane-L is preferable only when segmentation quality is prioritized over speed.

The throughput reduction from 259.42 to 104.04 FPS is 59.9%, which is substantial even though the resulting rate remains above real-time requirements on the evaluation GPU. This measurement does not establish suitability for resource-constrained edge processors or microcontrollers. Table 4 shows that single-level HSCM placements provide higher-throughput alternatives with smaller accuracy gains, offering one architecture-level deployment trade-off. Future edge-oriented work should evaluate integer quantization, structured pruning, knowledge distillation, operator fusion, and hardware-aware implementations of window partitioning and shifted-window attention. These options require direct measurement on the target device because latency, memory use, and operator efficiency are hardware dependent.

The absolute IoU values should also be interpreted carefully. Lane markings are extremely sparse and thin, so a small lateral shift or thickness mismatch can strongly reduce pixel-level IoU. Moreover, the BDD100K setting used here involves thicker training masks and thinner validation masks, which makes the overlap metric conservative. Therefore, an IoU in the mid-30% range should not be compared directly with IoU values for large semantic objects such as road, sky, or vehicles. These results support relative improvement under the BDD100K lane-mask protocol, but they are not sufficient by themselves to claim safety readiness for autonomous driving.

### Evaluation scope, limitations, and failure modes

The comparative results should be read within the evaluation scope of this paper. BDD100K is the main benchmark, whereas TuSimple is used only as an out-of-domain test set under a derived pixel-level protocol. The TuSimple numbers are therefore not official TuSimple lane-detection scores. They indicate cross-domain transfer of binary lane-mask predictions after training on BDD100K, where HSCM-Lane-S, HSCM-Lane-M, and HSCM-Lane-L obtain 27.2%, 27.6%, and 27.6% lane IoU without target-domain fine-tuning. Similarly, comparisons with previously published models should be interpreted cautiously when the baselines are cited from the literature rather than reproduced in a single unified training pipeline.

The condition-wise analysis reveals the main limitations of HSCM-Lane. The model is more reliable in common city-street, residential, and highway scenes than in rare or visually ambiguous contexts. Performance decreases under rain, snow, fog, weak illumination, and nighttime reflections because the lane markings lose contrast and often become indistinguishable from the road surface. Parking lots, tunnels, and gas-station scenes are also difficult because their markings can be short, crossing, fragmented, or visually similar to curbs, pavement symbols, and artificial lighting. These findings show that HSCM improves geometric continuity, but it does not remove errors caused by rare layouts, severe visibility loss, or annotation ambiguity.

### Practical implications and future extensions

From a practical perspective, HSCM-Lane is most useful as a specialized lane-mask branch for ADAS and autonomous-driving perception pipelines. Its advantages are a simple ResNet-based encoder-decoder structure, explicit multi-level context modeling, a lightweight decoder, and controlled HSCM-Lane-S/M/L operating points. The dense lane mask can provide geometric cues for lane keeping, road-structure inference, local trajectory planning, or downstream lane fitting. At the same time, the model should not be treated as a complete driving-perception system because it does not output lane instances, does not model temporal consistency, and does not jointly reason about objects, drivable area, or vehicle control.

At a broader system level, NavigScene [27] shows how local sensor representations can be combined with beyond-visual-range navigation guidance for perception, prediction, and planning. HSCM-Lane addresses only the local lane-mask component and does not perform navigation reasoning or trajectory generation. Its binary mask should therefore be viewed as a geometry-focused input that could be integrated with navigation-guided models, not as a complete end-to-end autonomous-driving stack.

Future work should therefore extend the current model in several directions. First, temporal modeling across video frames should be added to reduce flickering and improve lane continuity under occlusion. Second, binary masks should be connected to lane-instance reconstruction or curve fitting so that the output can be used more directly by planning modules. Third, multimodal inputs, drivable-area cues, object context, uncertainty estimation, and failure detection should be investigated for difficult weather, night scenes, and rare layouts. Finally, the method should be evaluated not only by pixel-level metrics but also by downstream planning impact and safety-oriented validation. The same design principle may also be useful for other thin-structure segmentation tasks, such as road markings, cracks, rails, or curvilinear structures, where local detail and long-range continuity must be modeled together.

## Conclusion

In this paper, we proposed HSCM-Lane, a ResNet-based encoder-decoder architecture for pixel-wise binary lane segmentation. The same design was evaluated under three backbone-capacity configurations, denoted as HSCM-Lane-S, HSCM-Lane-M, and HSCM-Lane-L. The core idea is to insert shifted-window context modeling at the 1/4, 1/8, and 1/16 encoder feature levels, so that context-enhanced features are propagated through the encoder and reused by a lightweight decoder with projected static-sum fusion.

On BDD100K, HSCM-Lane-S achieves 34.44 ± 0.13% lane IoU, 65.00 ± 0.19% lane Recall, and 82.12 ± 0.08% Balanced Accuracy over five seeds, improving on the no-HSCM counterpart. HSCM-Lane-M and HSCM-Lane-L further improve lane IoU to 34.86 ± 0.05% and 34.98 ± 0.04%, respectively, but with higher computational cost. Under the derived TuSimple pixel-level protocol, the three variants also show reasonable out-of-domain transfer without target-domain fine-tuning.

Overall, the results show that combining the local inductive bias of CNNs with hierarchical attention is an effective, lightweight, and suitable design direction for lane segmentation in diverse traffic scenarios. The method improves lane-mask continuity and reduces missed detections, but further work is required before safety-critical deployment, especially on temporal consistency, lane-instance reconstruction, multimodal robustness, downstream integration, and safety-oriented validation.
